# Dysregulation of phosphatidylethanolamine metabolism associated with upregulation of PNPLA6 in high-fat diet/streptozotocin mice

**DOI:** 10.3389/fnut.2026.1799991

**Published:** 2026-07-07

**Authors:** Nao Inoue, Hsin-Jung Ho, Siddabasave Gowda B. Gowda, Xun-Zhi Wu, Miki Eguchi, Minato Masamura-Takeuchi, Hitoshi Chiba, Shu-Ping Hui

**Affiliations:** 1 Faculty of Health Sciences, Hokkaido University, Sapporo, Japan; 2 Graduate School of Global Food Resources, Hokkaido University, Sapporo, Japan; 3 Department of Nutrition, Sapporo University of Health Sciences, Sapporo, Japan

**Keywords:** glycerophosphorylethanolamine, liquid chromatography-tandem mass spectrometry, lysophosphatidylethanolamine, lysophospholipase, lysophospholipid, neuropathy target esterase

## Abstract

**Background:**

Dysregulation of lipid metabolism plays a key role in diabetes mellitus (DM). Phosphatidylethanolamine (PE) and its metabolites, lysophosphatidylethanolamine (LPE) and glycerophosphorylethanolamine (GPE), are involved in cell signaling and mitochondrial homeostasis. Disruption of PE metabolism is associated with insulin sensitivity and mitochondrial dysfunction. However, the mechanism underlying alterations in PE metabolites, especially LPE, in DM remains unclear.

**Methods:**

Six-week-old male C57BL/6N mice were divided into three groups: control (regular diet, *n* = 8), high-fat diet (HFD, *n* = 8), and HFD combined with streptozotocin treatment (HFD/STZ, *n* = 6). Lipids were extracted from plasma, kidney, and liver samples using a single-phase method, followed by targeted (LPE 16:0, 18:0, 18:1, 18:2, 20:4, 20:5, and 22:6) and non-targeted liquid chromatography-tandem mass spectrometry. Protein expression was analyzed by Western blotting.

**Results:**

In the plasma of HFD/STZ mice, the levels of combined LPEs were increased in parallel with PE levels, whereas most LPE species were significantly decreased in the kidneys. Interestingly, GPE levels were markedly increased in the plasma, kidneys, and liver of HFD/STZ mice. The LPE/GPE ratio in the plasma and kidney was negatively correlated with key indicators of DM, such as inflammation, glucose intolerance, and renal dysfunction. Furthermore, PNPLA6, a lysophospholipase that cleaves acyl chains from PE and LPE, was upregulated in the kidney of HFD/STZ mice.

**Conclusion:**

Upregulation of PNPLA6 was associated with the reduced LPE/GPE ratio, which may contribute to the progression of DM. This study provides new insights into DM pathology and therapeutic targets.

## 1 Introduction

Over the past few decades, the prevalence of diabetes mellitus (DM) has increased significantly worldwide (1–3). It is estimated that more than 20% of patients with type 2 DM develop renal disease (4–6). Hypertension, poor glycemic control, and dyslipidemia in patients with type 2 DM are reversible risk factors for kidney disease progression (7, 8). Diabetic nephropathy is a common microvascular complication of DM that leads to chronic kidney disease (9) and is clinically characterized by microalbuminuria and a progressive decrease in glomerular filtration rate (10). Dysregulation of lipid metabolism, such as elevated serum triacylglycerides, free fatty acids (FAs), and cholesterol, is linked to lipotoxicity and lipid accumulation in DM, which plays a significant role in the pathogenesis of DM and diabetic complications (9). However, the mechanisms underlying lipid dysfunction in the pathogenesis of DM remain to be elucidated.

Dysregulation of phospholipids is involved in the pathogenesis of type 2 DM (11). Phosphatidylcholine (PC) and phosphatidylethanolamine (PE) are the most prevalent phospholipids in mammalian cells, playing crucial roles in maintaining mitochondrial homeostasis and function (12). Previous studies have indicated that PC and its deacylated product, lysophosphatidylcholine (LPC), are associated with insulin resistance and impaired glucose tolerance in DM, with the underlying mechanisms elucidated (11, 13). Conversely, research on the fluctuations of PE metabolites, especially lysophosphatidylethanolamine (LPE), remains limited. PE is a major glycerophospholipid, with ethanolamine as the polar head group and two FAs as acyl chains. LPE is a deacylated product of PE, with one acyl chain, produced by phospholipases A1 and A2 (PLA1/2). PE is essential for complex III formation in mitochondria, and its deficiency affects the respiratory capacity and ATP production (14–16). LPE ameliorates mitochondrial dysfunction by recovering mitochondrial PE contents (16, 17). In recent years, research on the relationship between LPE and DM has been ongoing. On the one hand, LPE was decreased in the plasma of patients with cardiovascular disease accompanied by type 2 DM (18). On the other hand, plasma LPE levels were increased in patients with diabetic nephropathy (19). The LPE alterations in the plasma of patients with DM, including diabetic nephropathy, remain controversial. As mitochondrial dysfunction is described as a key event in the development of diabetic complications (20, 21), dysregulation of PE metabolism is thought to be associated with the pathology of DM.

To date, semi-quantitative methods have been used for the determination of LPEs because of the lack of optimal quantitative methods using authentic LPE standards. We previously developed a sensitive and efficient quantitative method for seven major LPEs (LPE 16:0, 18:0, 18:1, 18:2, 20:4, 20:5, and 22:6) using liquid chromatography-tandem mass spectrometry (LC-MS/MS) (22). In this study, we applied our well-established quantitative method to the plasma and organs of high-fat diet (HFD)/streptozotocin (STZ) mice. The combination of HFD and STZ injections in C57BL/6 mice, which are highly susceptible to the metabolic effects of HFD feeding (23), induces renal dysfunction accompanied by glucose intolerance and insulin resistance (24, 25). This study aimed to reveal the relationship between PE metabolites, including LPE, and the pathology of DM, and the mechanisms underlying PE metabolite alterations. To this end, we determined the PE metabolite profiles in the plasma, kidney, and liver of HFD/STZ mice and investigated the expression of genes and proteins related to the synthesis and catabolism of PE metabolites in HFD/STZ mice.

## 2 Materials and methods

### 2.1 Materials

LC-MS-grade methanol (MeOH) and acetonitrile were purchased from Kanto Chemical Co. Inc. (Tokyo, Japan). LC-MS-grade isopropanol and chloroform were acquired from FUJIFILM Wako Pure Chemical Corporation (Osaka, Japan) and Nacalai Tesque Inc. (Kyoto, Japan), respectively. Ammonium acetate (1 mol/L solution), ethylenediaminetetraacetic acid, and Tween 20 were obtained from Sigma-Aldrich (St. Louis, MO, Louis, MO, United States). 2,6-di-tert-butyl-p-cresol was obtained from Tokyo Chemical Industry Co., Ltd. (Tokyo, Japan). EquiSPLASH lipidomix, which was used as an internal standard (IS), was purchased from Avanti Polar Lipids, Inc. (AL, United States). 1-palmitoyl-2-hydroxy-*sn*-glycero-3-phosphoethanolamine (LPE 16:0), 1-stearoyl-2-hydroxy-*sn*-glycero-3-phosphoethanolamine (LPE 18:0), and 1-oleoyl-2-hydroxy-*sn*-glycero-3-phosphoethanolamine (LPE 18:1) were purchased from Avanti Polar Lipids, Inc. (AL, United States). Additionally, 1-linoleoyl-2-hydroxy-*sn*-glycero-3-phosphoethanolamine (LPE 18:2), 1-arachidonyl-2-hydroxy-*sn*-glycero-3-phosphoethanolamine (LPE 20:4), 1-eicosapentaenoyl-2-hydroxy-*sn*-glycero-3-phosphoethanolamine (LPE 20:5), and 1-docosahexaenoyl-2-hydroxy-*sn*-glycero-3-phosphoethanolamine (LPE 22:6) were synthesized in-house (26).

### 2.2 Animal experiments

All animal experiments were approved by the Animal Care Committee of Hokkaido University (approval protocol number: 22-0140). HFD/STZ mice were prepared according to a previous study with certain modifications (25). Six-week-old male C57BL/6 N mice were obtained from CLEA Japan, Inc. (Tokyo, Japan). The mice were housed at 22 ± 2 °C, with free access to water and food, under a 12/12-h light/dark cycle. Before the start of the experiments, mice were divided into three groups randomly. The control and HFD groups were fed a normal chow diet (CE-2, CLEA Japan, Inc., Tokyo, Japan) and an HFD comprising 60% fat of total calories and 5.24 kcal/g (HFD32, CLEA Japan, Inc., Tokyo, Japan) for 8 weeks, respectively. The HFD/STZ group was fed HFD for 8 weeks, followed by an injection of STZ (35 mg/kg, i.p., Fujifilm Wako Pure Chemical Corporation, Osaka, Japan) dissolved in saline (Otsuka Pharmaceutical Co., Ltd., Tokyo, Japan) for five consecutive days. Mice in the control (*n* = 8), HFD (*n* = 8), and HFD/STZ groups (*n* = 6) were sacrificed after 4 weeks of STZ treatment (Supplementary Figure 1). Blood samples were collected by cardiac puncture and transferred into tubes containing heparin sodium (Mochida Pharmaceutical Co., Ltd., Tokyo, Japan) immediately following anesthesia with 2% isoflurane (Fujifilm Wako Pure Chemical Corporation, Osaka, Japan), with no intentional delay, to minimize pain and distress, and stored at −80 °C. The kidney and liver samples were promptly snap-frozen in dry ice and stored at −80 °C.

### 2.3 Histology

The sections of the kidney were fixed with 10% formalin and embedded in paraffin. Hematoxylin-Eosin and Periodic acid-Schiff staining were performed at the Sapporo General Pathology Laboratory (Sapporo, Japan).

### 2.4 Measurement of biological indices

All biological indices of the plasma samples collected after anesthesia at the end of 13 weeks were assessed using commercial kits, following the manufacturer’s instructions. Fasting blood glucose (FBG) levels were quantified using a biosensor-based chip (LAB Gluco, Research & Innovation Japan Inc., Chiba, Japan). Plasma creatinine (CRE) levels were determined using a creatinine colorimetric assay kit (Cayman Chemical, MI, United States). The urine albumin-to-creatinine ratio (UACR) was calculated based on urine albumin and CRE levels. Urine albumin levels were measured using an LBIS Mouse Albumin ELISA kit (Fujifilm Wako Pure Chemical Corporation, Osaka, Japan) with urine samples diluted 100-fold. Urine CRE was measured using a creatinine (urinary) colorimetric assay kit (Cayman Chemical, MI, United States) with urine samples diluted 20-fold. Alanine aminotransferase (ALT) and aspartate aminotransferase (AST) levels were measured using an Alanine Transaminase Activity Assay Kit (Cayman Chemical, MI, United States) and an Aspartate Aminotransferase Activity Assay Kit (Cayman Chemical, MI, United States), respectively.

### 2.5 Lipid extraction

Lipids were extracted from the samples using a single-phase method as previously described, with certain modifications (22). In brief, 100 mg of organ samples were weighed, followed by the addition of 1 mL of cold MeOH and homogenization using a Bead Mill 4 Homogenizer (Fisherbrand, Pittsburgh, PA, United States). The supernatant (50 μL) was combined with 100 μL of MeOH and 50 μL of IS (EquiSPLASH lipidomix [0.2 μg/mL]) in MeOH. For plasma samples, 10 μL of plasma was added to 200 μL of MeOH containing the IS. Subsequently, 100 μL of chloroform and 20 μL of Milli-Q water were added to the mixture. Following vortex mixing and centrifugation, the single-phase centrifuge was dried under vacuum at 4 °C. The dried lipids were dissolved in 100 μL of MeOH (final IS concentration: 0.1 μg/mL) and centrifuged at 4 °C for 10 min to remove any insoluble materials. MeOH used for extraction contained 0.01% (w/v) 2,6-di-tert-butyl-*p*-cresol to prevent oxidation. The prepared samples were subjected to targeted and non-targeted LC-MS/MS analysis.

### 2.6 Targeted LC-MS/MS analysis of LPE species

LC-MS/MS analysis was conducted as described previously (22). LC separation was performed using a high-performance liquid chromatography system (Shimadzu, Kyoto, Japan) equipped with a Hypersil GOLD column (50 × 2.1 mm, 5.0 μm, Thermo Fisher Scientific Inc., MA, United States) maintained at 45 °C. The sample tray temperature was kept at 4 °C. Five microliter of the sample was injected into the system. Chromatographic separation employed a mobile phase consisting of 60% Milli-Q water, 20% MeOH, and 20% acetonitrile with 5 mmol/L aqueous ammonium acetate containing 500 nmol/L ethylenediaminetetraacetic acid (solvent A), and isopropanol (solvent B). The gradient at a flow rate of 0.4 mL/min was applied: 0–1 min (80% A, 20% B); 1–3 min (40% A, 60% B); 3–5 min (40% A, 60% B); 5–6 min (0% A, 100% B); 6–7 min (0% A,100% B); and 7–10 min (80% A, 20% B).

A TSQ Quantum Access mass spectrometer (Thermo Fisher Scientific, Inc., Waltham, MA, United States) was operated in electrospray ionization negative ion mode. The optimized ion source parameters were as follows: spray voltage, 3,000 V; vaporizer temperature, 300 °C; capillary temperature, 200 °C; sheath gas (nitrogen) pressure, 30 psi; ion sweep gas (nitrogen) pressure, 4 psi; and auxiliary gas (nitrogen) pressure, 35 psi. The single reaction monitoring channels by collision-induced dissociation obtained for each LPE species are listed in Supplementary Table 1. The recovery rates for each LPE species in the kidneys and livers are provided in Supplementary Table 2. The recovery rates of the plasma samples were reported in a previous study (22). The linearity, limits of detection, and limits of quantification of each LPE species are presented in Supplementary Table 3.

### 2.7 Non-targeted LC-MS/MS analysis

Non-targeted lipidomic analysis was conducted utilizing a high-performance liquid chromatography system (Shimadzu, Kyoto, Japan) in conjunction with an LTQ-Orbitrap mass spectrometer (Thermo Fisher Scientific Inc., MA, United States) operating in the electrospray ionization negative and positive ion mode. A 10 μL aliquot of the sample was introduced into the instrument for analysis. LC separation was achieved on an Atlantis T3 C18 column (2.1 mm × 150 mm, 3 μm, Waters, MA, United States) at a flow rate of 0.2 mL/min at 4 °C, employing a mobile phase consisting of 10 mM ammonium acetate aqueous solution (A), isopropanol (B), and MeOH (C). The gradient elution program for the negative ion mode was set as follows: 0–1 min (30% B, 35% C), 1–9 min (75% B, 15% C), 9–21 min (82.5% B, 15% C), 21–25 min (95% B, 5% C), 25–26 min (30% B, 35% C) and kept this ratio until 30 min. For the positive ion mode, the gradient elution program was set as follows: 0–1 min (30% B and 35% C), 1–9 min (82.5% B and 15% C), 9–15 min (95% B and 5% C), 15–25 min (95% B and 5% C), 25–26 min (30% B and 35% C) and kept this ratio until 30 min.

The MS conditions were established as described previously (27). The raw data were processed with a mass tolerance of 5.0 ppm using MS-DIAL 4.9 (28) and Xcalibur 2.2 (Thermo Fisher Scientific, MA, United States). The quantification of the analyte in the sample was determined by calculating the peak area ratios of the analyte to the IS and multiplying them by the amount of added IS. The analyte quantities were subsequently corrected based on the organ weight or plasma volume.

### 2.8 Gene expression

RNA was extracted from kidney samples using a NucleoSpin kit (TaKaRa Bio Inc., Shiga, Japan) and subsequently reverse-transcribed into cDNA using a ReverTra Ace qPCR RT Master Mix with gDNA remover (TOYOBO, Osaka, Japan), following the manufacturer’s protocol. The gene expressions of *Patatin like phospholipase domain containing 6*–*9* (*Pnpla6*–*9*), *Lysophosphatidylcholine acyltransferase 3/4* (*Lpcat3/4*), and *Lysophosphatidylethanolamine acyltransferase 1* (*Lpeat1*) were assessed via quantitative polymerase chain reaction using a THUNDERBIRD SYBR qPCR Mix (TOYOBO, Co., Ltd., Osaka, Japan). The primer sequences are listed in Supplementary Table 4. All assays were conducted using a CFX Connect Real-Time PCR Analysis System (Bio-Rad Laboratories, Hercules, CA, United States). The expression levels of the target genes were quantified using the 2^−ΔΔCt^ method and normalized to *β-actin* expression levels.

### 2.9 Western blotting

Following homogenization of the kidney samples, protein levels were quantified using the Pierce BCA Protein Assay Kit (Thermo Fisher Scientific, Waltham, MA). Subsequently, equal protein quantities (30 μg) were resolved on a 5–20% SDS-PAGE gel (ATTO Corporation, Tokyo, Japan) and transferred onto a 0.2 μm polyvinylidene difluoride membrane (Millipore, Bedford, MA, United States). The membrane was blocked to prevent nonspecific binding using 5% skim milk in Tris-buffered saline with 0.1% Tween 20 for 1 h at room temperature. Subsequently, the membranes were incubated overnight at 4 °C with primary antibodies specific to neuropathy target esterase, also known as PNPLA6 (1:1,000, sc-271049, Santa Cruz Biotechnology, Dallas, TX, United States), PNPLA7 (1:1,000, FLJ00415, ProteinExpress Co., Ltd., Chiba, Japan), and β-actin (1:1,000, M177-3, MBL Co., Ltd., Nagoya, Japan). Following washing with Tris-buffered saline with 0.1% Tween 20, the membranes were incubated with secondary antibodies (1:10,000) for 1 h at room temperature and subsequently reacted with horseradish peroxidase substrate (WSE-7120S/7120L EzWestLumi Plus, ATTO Corporation, Tokyo, Japan). Stained bands were visualized using an enhanced chemiluminescence detection system (ATTO Corporation, Tokyo, Japan). Images were captured using a CCD system (ChemiDoc MP Imaging System, Bio-Rad, Hercules, CA, United States). The relative expression levels of each protein were normalized to that of β-actin.

### 2.10 Statistics

GraphPad Prism 8.0.1 (GraphPad Software Inc., La Jolla, CA, United States) was used for the analysis. The non-parametric Kruskal–Wallis test was used for all statistical analyses. Statistical significance was set at *p* < 0.05. Outliers were identified using the interquartile range method when necessary and excluded from the analysis. Correlation analysis was performed by calculating the Spearman correlation coefficient between metabolite levels and biological indices. Receiver operating characteristic curve analysis was used to determine the area under the curve (AUC) and 95% confidence interval (CI). Heatmap visualization was performed by MetaboAnalyst 6.0^1^ using auto-scaled data.

## 3 Results

### 3.1 Characteristics of the HFD/STZ model mice

In this study, HFD combined with STZ injection was used to induce key features of DM for 13 weeks. Consistent with previous studies (24, 25), HFD/STZ mice exhibited glucose intolerance, with an approximate value of 250 mg/dL (Supplementary Table 5), the threshold for a diagnosis of type 2 DM mice (29). Indicators of renal dysfunction, such as plasma CRE and UACR, were significantly increased in the HFD/STZ group compared with those in the control group. AST, a marker of systemic inflammation, was significantly higher in the HFD/STZ group than in the control group. Although there was no significant difference in ALT, a marker of liver injury, it tended to increase in the HFD/STZ group compared with the control group. Moreover, histological analysis revealed the expansion of the mesangium in the kidney of HFD/STZ mice (Supplementary Figure 2). The mesangial expansion is a hallmark of diabetic nephropathy and serves as a central mechanism for renal dysfunction (30). These results indicate that HFD/STZ mice mimic features of DM accompanied by renal dysfunction.

### 3.2 LPE alterations in the plasma and organs of HFD/STZ model mice

**Figure 1:**
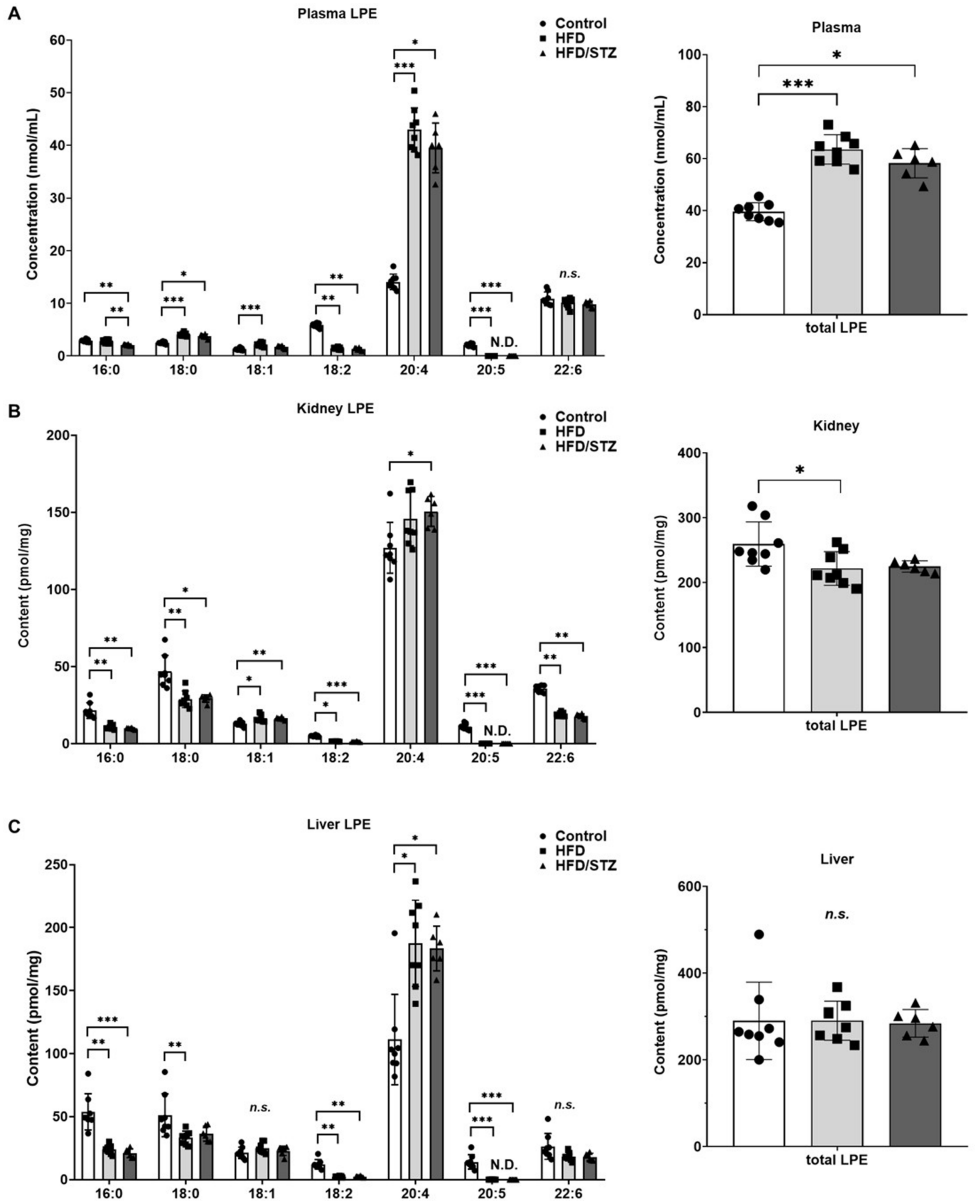
Absolute LPE levels in plasma **(A)**, kidney **(B)**, and liver **(C)** of the control, HFD, and HFD/STZ mice. Results are expressed as mean ± standard deviation. *p < 0.05, **p < 0.01, ***p < 0.001. n.s., not significant. HFD, high-fat diet; LPE, lysophosphatidylethanolamine; N.D., not detected; STZ, streptozotocin.

To determine the absolute LPE levels in the plasma and central organs of lipid metabolism, including the kidney and liver, LPEs were measured using our quantitative method (22). Plasma LPE 18:0 and 20:4 were significantly increased in both HFD and HFD/STZ groups compared with the control group, whereas LPE 16:0, 18:2, and 20:5 were significantly decreased in the plasma of HFD/STZ mice compared with the control (Figure 1A). According to the increase in LPE 20:4, the total plasma LPE (combined seven LPEs) was significantly increased in both HFD and HFD/STZ groups. In the kidneys of the HFD/STZ group, except for LPE 18:1 and 20:4, most LPEs were significantly decreased compared with the control (Figure 1B). Although the levels of total kidney LPE tended to decrease in the HFD/STZ group compared with the control, the difference was not statistically significant (*p* = 0.146). LPE alteration in the liver of HFD/STZ mice showed a trend similar to that in the kidneys of HFD/STZ mice. However, there was no significant difference in the total liver LPE levels among all groups, and the alterations were less pronounced than those observed in the kidney (Figure 1C). The detailed LPE levels in the samples are listed in Supplementary Tables 6, 7. The receiver operating characteristic curve analysis demonstrated that plasma LPE 16:0, plasma LPE 18:1, and kidney LPE 18:2 were effective in distinguishing between HFD and HFD/STZ groups (Supplementary Table 8). Nonetheless, the correlation analysis revealed a lack of consistent patterns regarding the types of LPE species and their relationships with biological indices, thereby complicating the evaluation of DM pathology using LPE alone (Supplementary Table 9).

### 3.3 Dysregulation of phospholipid metabolism in HFD/STZ model mice

**Figure 2:**
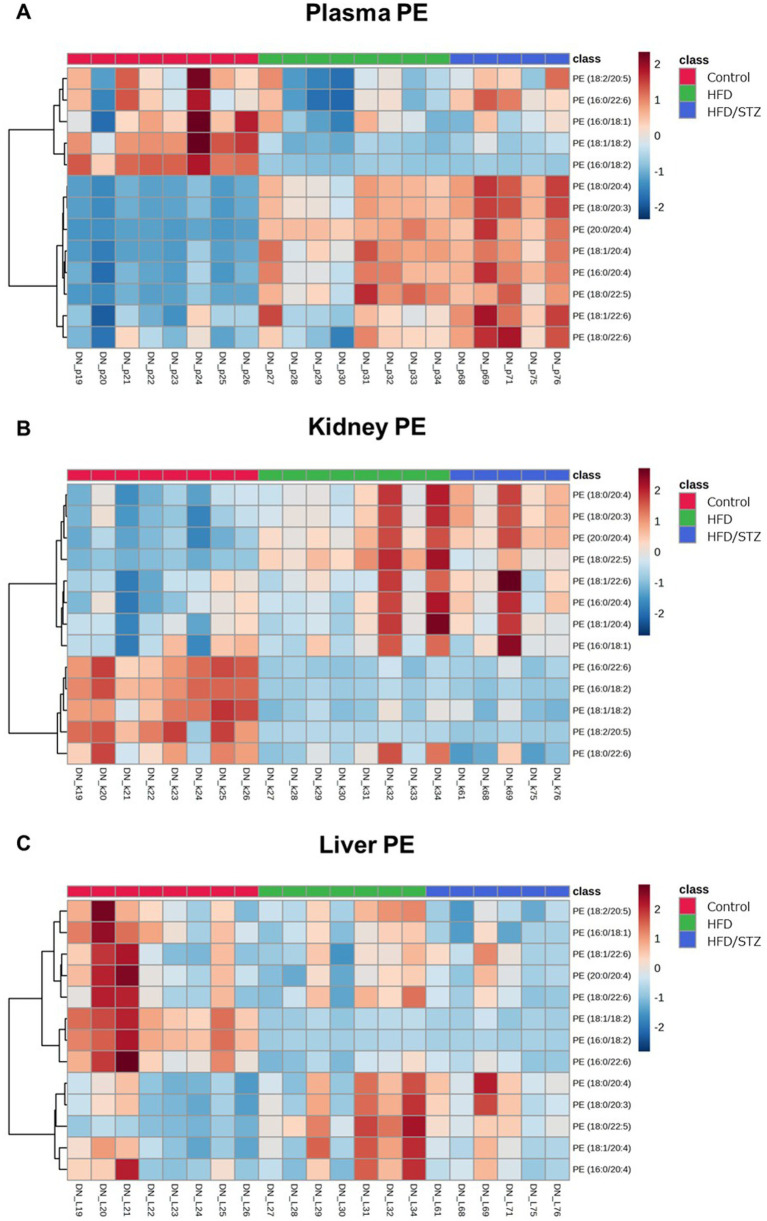
Heatmaps of PE species in the plasma **(A)**, kidney **(B)**, and liver **(C)** samples. HFD, high-fat diet; PE, phosphatidylethanolamine; STZ, streptozotocin.

**Figure 3:**
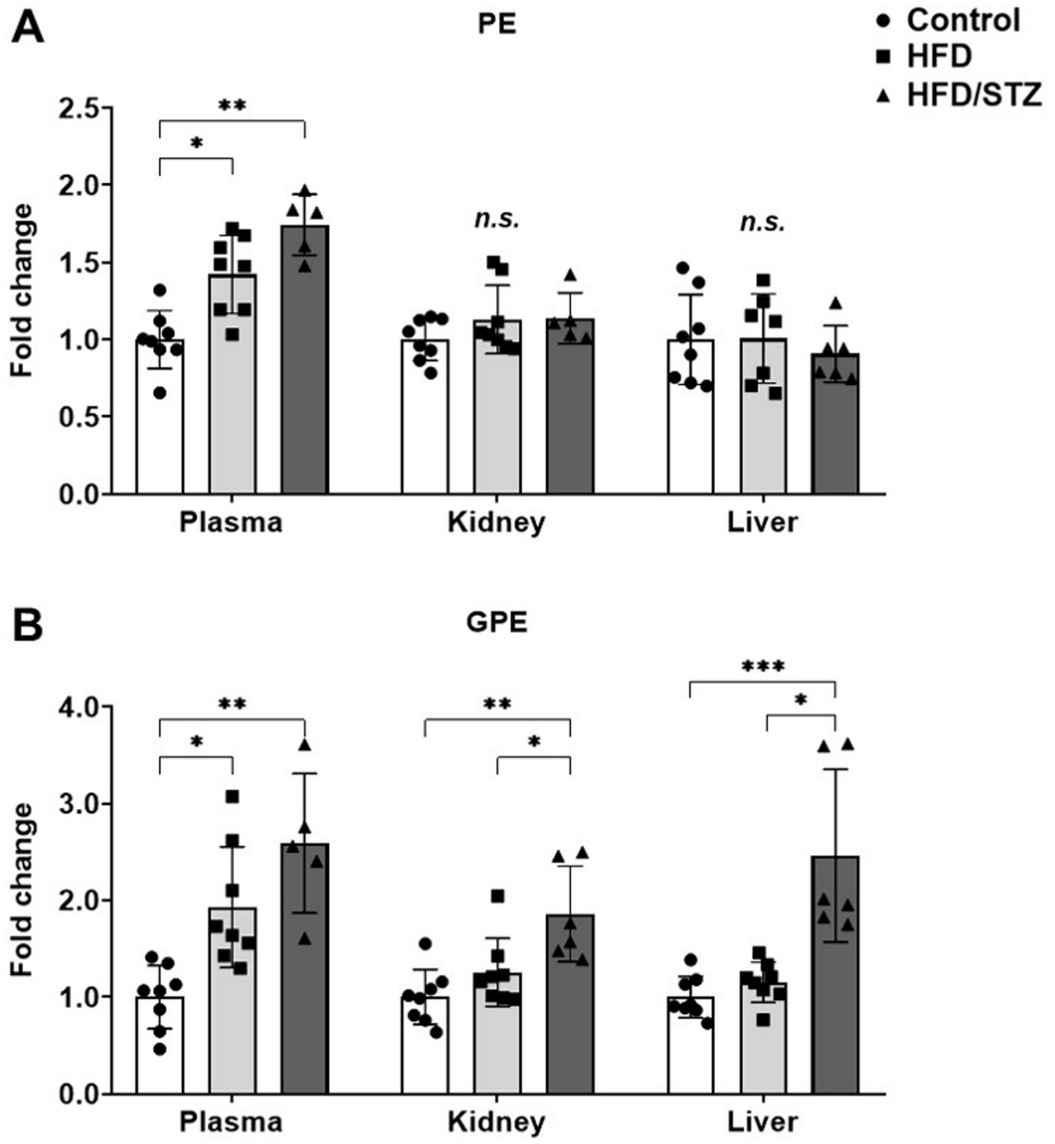
Relative abundance of total PE **(A)** and GPE **(B)** in the plasma, kidney, and liver samples. Results are expressed as mean ± standard deviation. *p < 0.05, **p < 0.01, ***p < 0.001, n.s., not significant. GPE, glycerophosphorylethanolamine; HFD, high-fat diet; PE, phosphatidylethanolamine; STZ, streptozotocin.

To achieve a comprehensive understanding of PE metabolites, we examined the alterations in PE and its deacylated product, glycerophosphorylethanolamine (GPE) without acyl chains. Thirteen PE species were identified at the MS^2^ level using non-targeted LC-MS/MS (Figure 2). PEs primarily containing FA 18:0 and 20:4 as acyl chains in the plasma and liver exhibited an increase in both HFD and HFD/STZ groups compared with the control. In contrast, PEs containing FA 16:0, 18:2, and 20:5 as acyl chains in the plasma and liver were decreased in both HFD and HFD/STZ groups. In the kidney, PEs primarily containing FA 20:4 as acyl chains were increased in both HFD and HFD/STZ groups; PEs containing FA 16:0, 18:2, 20:5, and 22:6 were decreased. PEs containing FA 18:1 generally increased in the kidney of HFD and HFD/STZ groups, whereas PE (18:1/18:2) was decreased, and a consistent interpretation could not be obtained. Plasma PE levels were significantly increased in both HFD and HFD/STZ groups compared with the control, whereas there was no significant difference in the levels of PE in both the kidney and liver among all groups (Figure 3A). Interestingly, GPE levels in the plasma, kidney, and liver were significantly increased in both HFD and HFD/STZ groups compared with the control (Figure 3B). The detailed levels of PE and GPE in samples are listed in Supplementary Tables 10–12. Taken together, the degradation of LPE to GPE was enhanced in HFD/STZ mice. In particular, GPE accumulation was observed in the kidney and liver of the HFD/STZ mice without significant changes in the total PE levels.

**Figure 4:**
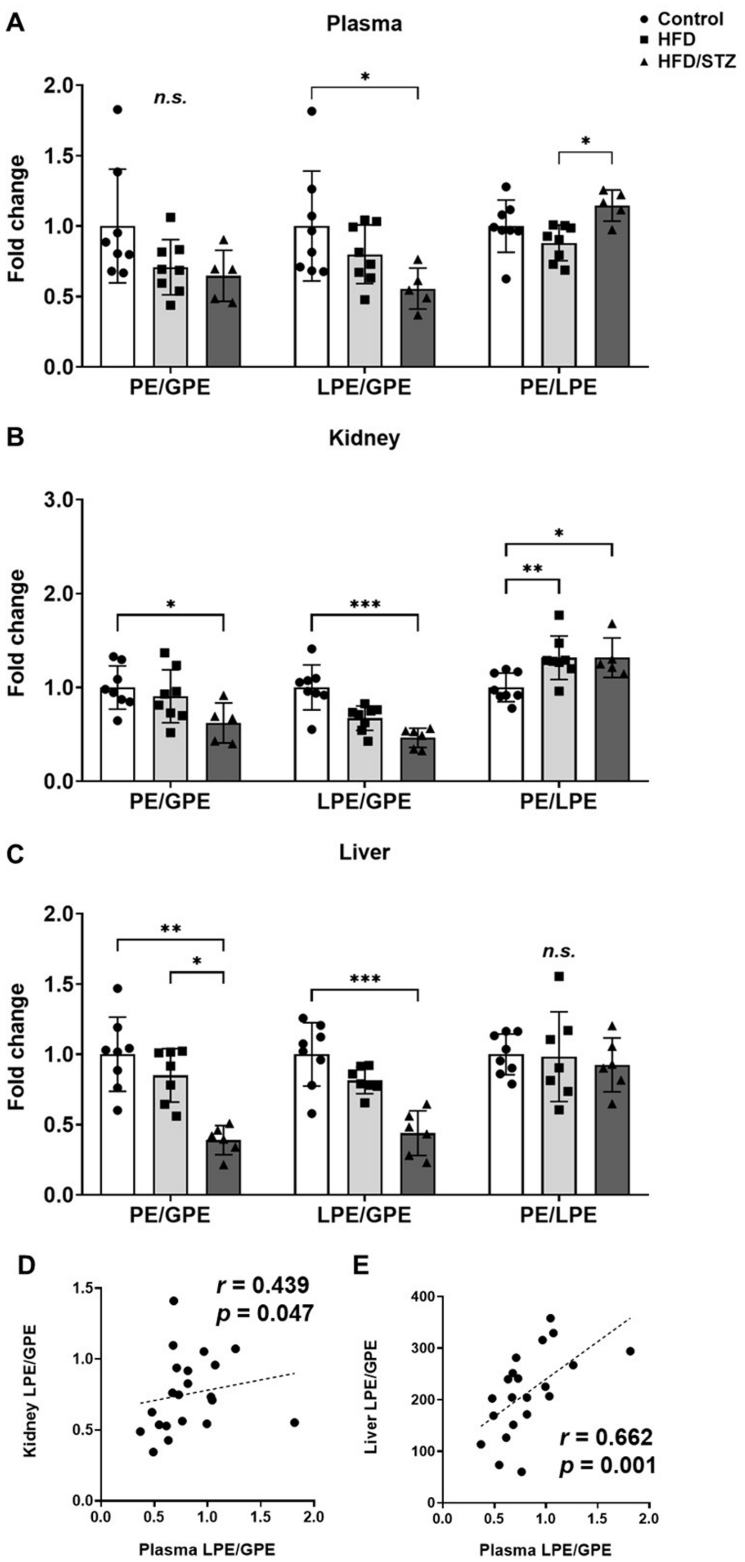
Alterations in the ratio of PE metabolites in the plasma, kidney, and liver samples. Comparisons of the ratio of PE metabolites in the plasma **(A)**, kidney **(B)**, and liver **(C)** among the control, HFD, and HFD/STZ groups (mean ± standard deviation). Correlation analysis of the LPE/GPE ratio between the plasma and kidney **(D)** or the plasma and liver **(E)**. *p < 0.05, **p < 0.01, ***p < 0.001, n.s., not significant. GPE, glycerophosphorylethanolamine; HFD, high-fat diet; LPE, lysophosphatidylethanolamine; PE, phosphatidylethanolamine; STZ, streptozotocin.

We investigated the ratio of PE metabolites, which is associated with membrane lipid degradation and metabolic stress (31–33), to evaluate the dysregulation of PE metabolism in the HFD/STZ mice. The plasma LPE/GPE ratio was significantly decreased in the HFD/STZ group compared with the control (Figure 4A), showing an AUC of 0.925 for the control vs. HFD/STZ group (Table 1) and negative correlations with AST, FBG, and CRE with low *p*-values (Table 2). In the kidney of the HFD/STZ group, both the PE/GPE and LPE/GPE ratios were significantly decreased, while the PE/LPE ratio was significantly increased (Figure 4B). Notably, when correlations with biological indices were examined across the entire dataset encompassing the control, HFD, and HFD/STZ groups, the kidney LPE/GPE ratio demonstrated strong negative correlations with AST, FBG, CRE, and UACR, with low *p*-values (Table 2). Furthermore, the kidney LPE/GPE ratio showed AUC of 0.906, 0.979, and 0.896 for control vs. HFD, control vs. HFD/STZ, and HFD vs. HFD/STZ, respectively (Table 1). In the liver of the HFD/STZ group, the PE/GPE and LPE/GPE ratios were significantly decreased compared with the control, showing AUC values exceeding 0.9 for control vs. HFD/STZ or HFD vs. HFD/STZ (Figure 4C and Table 1). As shown in Figure 4D, correlation analysis revealed a positive correlation between the plasma and kidney LPE/GPE ratios (*r* = 0.439, *p* = 0.047). Moreover, a positive correlation between the plasma and liver LPE/GPE ratios was observed, as shown in Figure 4E (*r* = 0.662, *p* = 0.001). Although it would be necessary to assess the association between inflammation and dysregulation of PE metabolites using more appropriate inflammatory markers in addition to AST, these findings nevertheless suggest that the LPE/GPE ratio can reflect diabetic status, such as inflammation, glucose intolerance, and renal dysfunction.

**Table 1:** Results of the receiver operating characteristic curve analysis.

Tissues	Lipid species	AUC (95% CI)		
		Control vs. HFD	Control vs. HFD/STZ	HFD vs. HFD/STZ
Plasma	PE	0.922** (0.791–1.000)	1.000** (1.000–1.000)	0.850* (0.627–1.000)
	LPE	1.000*** (1.000–1.000)	1.000** (1.000–1.000)	0.750 (0.487–1.000)
	GPE	0.969** (0.892–1.000)	1.000** (1.000–1.000)	0.750 (0.472–1.000)
	PE/GPE	0.719 (0.463–0.975)	0.775 (0.505–1.000)	0.525 (0.180–0.870)
	LPE/GPE	0.672 (0.397–0.947)	0.925* (0.766–1.000)	0.825 (0.584–1.000)
	PE/LPE	0.703 (0.430–0.976)	0.775 (0.508–1.000)	0.925* (0.766–1.000)
Kidney	PE	0.609 (0.316–0.903)	0.650 (0.338–0.962)	0.600 (0.286–0.914)
	LPE	0.797* (0.565–1.000)	0.896* (0.720–1.000)	0.625 (0.290–0.961)
	GPE	0.679 (0.396–0.961)	0.958** (0.858–1.000)	0.976** (0.904–1.000)
	PE/GPE	0.625 (0.337–0.913)	0.875* (0.680–1.000)	0.850* (0.628–1.000)
	LPE/GPE	0.906** (0.727–1.000)	0.979** (0.915–1.000)	0.896* (0.720–1.000)
	PE/LPE	0.938** (0.809–1.000)	0.925* (0.766–1.000)	0.550 (0.205–0.895)
Liver	PE	0.578 (0.281–0.875)	0.542 (0.226–0.857)	0.625 (0.315–0.935)
	LPE	0.578 (0.281–0.875)	0.604 (0.289–0.919)	0.542 (0.225–0.858)
	GPE	0.719 (0.453–0.984)	1.000** (1.000–1.000)	1.000** (1.000–1.000)
	PE/GPE	0.714 (0.441–0.987)	1.000** (1.000–1.000)	1.000** (1.000–1.000)
	LPE/GPE	0.786 (0.518–1.000)	0.979** (0.915–1.000)	1.000** (1.000–1.000)
	PE/LPE	0.554 (0.229–0.878)	0.604 (0.282–0.926)	0.500 (0.169–0.831)

*p < 0.05, **p < 0.01, ***p < 0.001. AUC, area under the curve; CI, confidence interval; GPE, glycerophosphorylethanolamine; HFD, high-fat diet; LPE, lysophosphatidylethanolamine; PE, phosphatidylethanolamine; STZ, streptozotocin.

**Table 2:** Correlation analysis between PE metabolites and biological indices.

Tissues	Lipid species	AST	ALT	FBG	CRE	UACR
Plasma	PE	0.710**	0.653*	0.565*	0.681*	0.604**
	LPE	0.714**	0.893***	0.450	0.367	0.507*
	GPE	0.789**	0.622*	0.640*	0.610*	0.690**
	PE/GPE	−0.574*	−0.389	−0.541*	−0.291	−0.348
	LPE/GPE	−0.727**	−0.490	−0.559*	−0.637*	−0.317
	PE/LPE	−0.029	−0.130	0.015	0.500	0.001
Kidney	PE	0.037	−0.103	0.480	0.291	0.286
	LPE	−0.757**	−0.811***	−0.259	−0.310	−0.382
	GPE	0.371	0.136	0.713**	0.579*	0.689**
	PE/GPE	−0.244	−0.160	−0.431	−0.357	−0.496*
	LPE/GPE	−0.650*	−0.482	−0.756**	−0.645*	−0.811****
	PE/LPE	0.613*	0.560*	0.537	0.385	0.641**
Liver	PE	−0.235	−0.305	0.055	0.058	−0.024
	LPE	−0.050	0.046	0.477	−0.029	−0.023
	GPE	0.475	0.229	0.776**	0.741**	0.653**
	PE/GPE	−0.692**	−0.503	−0.719**	−0.652*	−0.577*
	LPE/GPE	−0.507	−0.225	−0.613*	−0.772**	−0.623**
	PE/LPE	−0.371	−0.530	−0.205	0.003	−0.090

*p < 0.05, **p < 0.01, ***p < 0.001, ****p < 0.0001. ALT, alanine aminotransferase; AST, aspartate aminotransferase; CRE, creatinine; FBG, fasting blood glucose; GPE, glycerophosphorylethanolamine; HFD, high-fat diet; LPE, lysophosphatidylethanolamine; PE, phosphatidylethanolamine; STZ, streptozotocin; UACR, urine albumin-to-creatinine ratio.

Alterations in PC metabolites, including LPC and glycerophosphorylcholine (GPC), were also confirmed. In plasma, the levels of PC and LPC were significantly increased in both HFD and HFD/STZ groups compared with the control (Supplementary Figure 3). Plasma GPC was significantly increased in the HFD group compared with the control, but no significant difference was observed between the control and HFD/STZ groups. The PC metabolites in the kidney and liver showed similar behavior. In both organs, PC levels were significantly decreased in both HFD and HFD/STZ groups compared with the control. On the contrary, LPC in the kidneys of the HFD and HFD/STZ group showed a significant increase compared with the control, and demonstrated an increasing trend in the liver. Furthermore, the levels of GPC in both the kidneys and liver of the HFD/STZ group were significantly increased compared with the control. As shown in Supplementary Figure 4, there were no significant differences in the plasma PC/GPC, LPC/GPC, or PC/LPC ratios among any of the groups. In the kidneys of the HFD/STZ mice, the PC/GPC ratio was significantly decreased compared with the control, and the LPC/GPC ratio was significantly decreased compared with the HFD group. In the liver, all ratios—PC/GPC, LPC/GPC, and PC/LPC—were significantly decreased in both HFD and HFD/STZ groups compared with the control. While the ratios of PC/GPC and LPC/GPC and GPC levels in the kidney and liver showed effective AUC values (above 0.8) for distinguishing HFD vs. HFD/STZ groups, plasma PC metabolites did not show effective AUC values between the two groups (Supplementary Table 13). The correlation between biological indices and PC metabolites in plasma and liver showed a pattern similar to that of PE metabolites (Supplementary Table 14). However, in the kidney, due to the increase in LPC, the correlations of the PC/LPC ratio with AST, ALT, and UACR were negative, in contrast to those of the kidney PE/LPE ratio. The detailed levels of PC metabolites in samples are listed in Supplementary Tables 15–17.

### 3.4 Upregulation of PNPLA6 is associated with the dysregulation of PE metabolism

**Figure 5:**
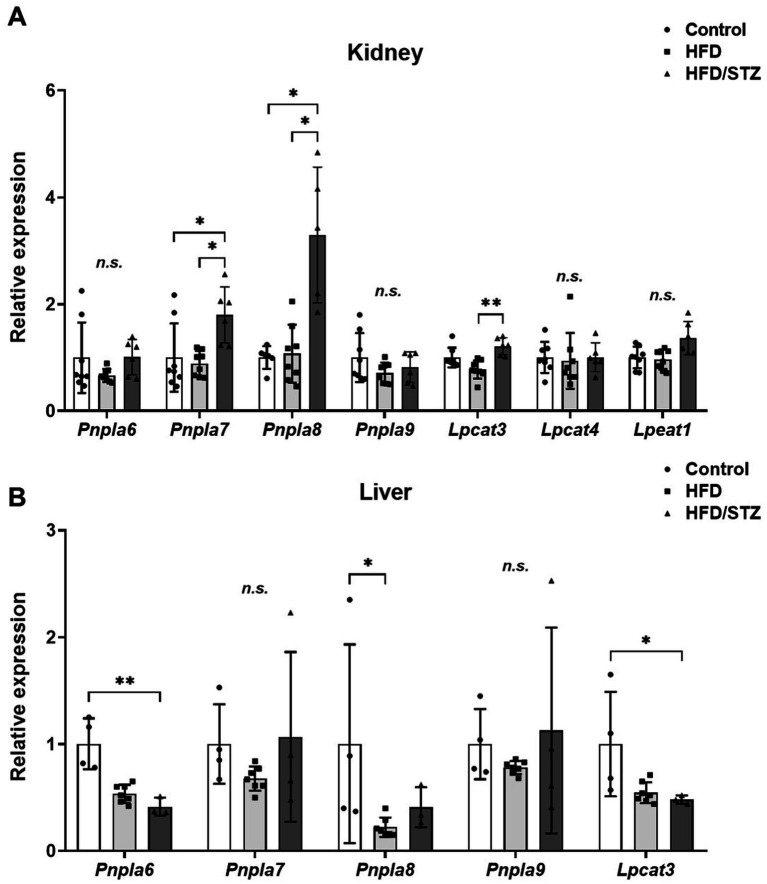
Relative expression levels of genes related to PE metabolism in the kidney **(A)** and liver **(B)**. Results are expressed as mean ± standard deviation. *p < 0.05, **p < 0.01, n.s., not significant. HFD, high-fat diet; Lpcat, lysophosphatidylcholine acyltransferase; LPE, lysophosphatidylethanolamine; Lpeat, lysophosphatidylethanolamine acyltransferase; Pnpla, patatin like phospholipase domain containing; STZ, streptozotocin.

To elucidate the mechanisms underlying alterations in PE metabolites, we assessed the gene expression levels of enzymes associated with PE and PC metabolism in the kidney and liver. In the kidney, the relative expressions of *Pnpla7* (lysophospholipase) and *Pnpla8* (PLA1/2) were significantly higher in the HFD/STZ group compared with those in the control and HFD groups (Figure 5A). *Lpcat3*, involved in PE and PC remodeling, was upregulated in the kidneys of the HFD/STZ group compared with those of the HFD group, but there was no significant difference between the control and HFD/STZ groups. No significant difference was observed in the expression of the other genes, such as *Pnpla6*, *Pnpla9*, *Lpcat4*, and *Lpeat1*. In the liver, *Pnpla6* and *Lpcat3* were significantly downregulated in the HFD/STZ group compared with the control, while no significant difference in the expression of *Pnpla7* and *Pnpla9* was observed across all groups (Figure 5B). *Pnpla8* was significantly downregulated in the liver of the HFD group compared with the control, whereas it did not show a significant difference between the control and the HFD/STZ group. The expression of *Lpcat4* and *Lpeat1* is low in the murine liver (34). In this study, *Lpcat4* and *Lpeat1* were not detected in the liver.

**Figure 6:**
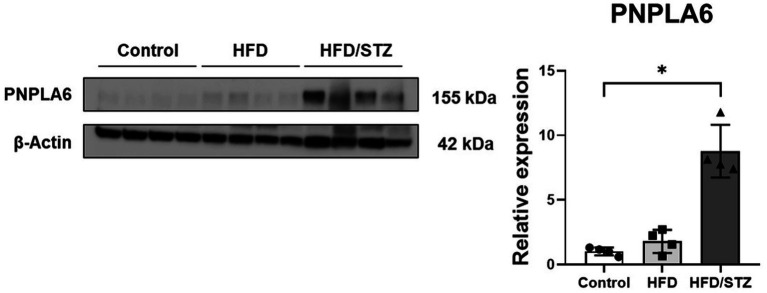
PNPLA6 protein expression in kidney samples. Results are expressed as mean ± standard deviation. *p < 0.05. HFD, high-fat diet; PNPLA6, patatin like phospholipase domain containing 6; STZ, streptozotocin.

The protein expression of PNPLA6 and PNPLA7 in the kidney was assessed to explore the association with LPE degradation. Interestingly, PNPLA6 protein expression was upregulated in the kidneys of the HFD/STZ group compared with the control (Figure 6). PNPLA7 protein in the kidney was below the detection limit by Western blotting with an antibody used in this study (data not shown). These results indicate that the upregulation of PNPLA6 is associated with the GPE accumulation in the kidney of the HFD/STZ group, which may contribute to the dysregulation of PE metabolism.

## 4 Discussion

Membrane PE metabolites are regulated by the orchestration of PLA and lysophospholipid acyltransferases (34). In the Kennedy pathway, one of the major pathways of PE synthesis, an ethanolamine is activated and transferred to a diacylglycerol molecule to generate PE. PLA (e.g., PNPLA8/9) and lysophospholipases (e.g., PNPLA6/7) cleave acyl chains from the glycerol backbone of PE to generate endogenous LPE and GPE (12). Conversely, lysophospholipid acyltransferases (e.g., LPCAT3, LPCAT4, and LPEAT1) acylate LPE to reconstruct PE through the Lands’ cycle. This constant turnover is vital for remodeling the membrane lipid composition, which regulates cell signaling and maintains organelle homeostasis (35).

We found that the accumulation of GPE in the kidneys of HFD/STZ mice was associated with the upregulation of PNPLA6 protein levels. In terms of gene expression, there was no significant difference in renal *Pnpla6* levels. Potential causes of this contradiction may include increased translation efficiency and disruption of protein homeostasis. PNPLA6 is degraded by autophagy and proteasome (36), and its dysregulation occurs under impaired glucose tolerance (37). Therefore, the accumulation of PNPLA6, resulting from the suppressed protein degradation, may be caused in the kidney of the HFD/STZ mice, even though there was no change in mRNA levels. However, the details remain unclear and warrant future investigation. PNPLA6 is a lysophospholipase that cleaves acyl chains at both *sn*-1 and *sn*-2 positions to generate LPE or GPE from PE and LPC or GPC from PC (38–41). PNPLA6 is widely distributed in murine tissues, mainly in the central nervous system and kidneys (38), and plays a crucial role in brain functions (42). However, there have been no reports on the role of renal PNPLA6 or the relationship between PNPLA6 and DM. To the best of our knowledge, this is the first report on the association between PNPLA6 and GPE accumulation in the HFD/STZ mice. Although we did not detect renal PNPLA7 protein in this study, which corresponds with a previous report that PNPLA7 is low expressed in the murine kidney (38), *Pnpla7*, as well as *Pnpla8*, were upregulated in the kidneys of HFD/STZ mice at the gene expression level. Previous research demonstrates the upregulation of PNPLA7 in metabolic dysfunction models, such as HFD mice and *db*/*db* mice (43). Furthermore, PNPLA7 and PNPLA8 coordinately regulate PC metabolism and, in part, PE metabolism (38). Therefore, their dysregulation may contribute to the accumulation of GPE, as well as GPC, in the kidney of HFD/STZ mice in this study.

Unlike the genetic alterations identified in the kidney, the expression level of *Pnpla6* was reduced in the liver of HFD/STZ mice. In previous research, the accumulation of GPE and GPC is reported to inhibit PLA2 activity, suggesting a negative feedback mechanism (32, 44). Therefore, a notable elevation in both GPE and GPC may contribute to the dysregulation of *Pnpla6* in the liver of HFD/STZ mice in this study. Furthermore, this study identified a decrease in *Lpcat3* expression in the livers of HFD/STZ mice with a reduction in PCs, which corresponds to the previous report that LPCAT3 expression in the liver diminishes under hepatic inflammation induced by HFD, consequently impairing phospholipid remodeling (45). Taken together, it is proposed that the dysregulation of these genes may contribute to the disruption of phospholipid metabolism in the liver of HFD/STZ mice.

The total plasma LPE levels were increased in the HFD/STZ group, which corresponds with previous reports on the elevation of LPE in the plasma of type 2 DM patients (19, 46). In contrast, several studies have reported that plasma or serum LPE, especially LPE 18:2, is decreased in patients with type 2 DM (18, 47). Consistent with a previous report (18), LPE 18:2 was decreased in the plasma of HFD/STZ mice. Furthermore, reductions in LPE 16:0 and 20:5 were also observed in this study. In the context of LPE alterations in the kidney, most LPEs were decreased in the HFD/STZ group and were negatively correlated with inflammation markers, which corresponds with reports on the anti-inflammatory effect of LPE (48, 49). Moreover, LPE is reported to improve the mitochondrial injury *in vivo* and *in vitro* (16, 17). Given the protective effects of LPE, dysregulation of renal LPE may be one of the factors that progress inflammation and renal dysfunction in DM. Nonetheless, it remains challenging to establish a consistent relationship between the behavior of individual LPE species in plasma or organs and the pathology of DM because of its complexity. Furthermore, the inflammation observed in HFD/STZ mice is likely influenced by STZ treatment, and the causal relationship between decreased LPE and inflammation needs to be clarified in future studies.

The GPE is a deacylated product of LPE that regulates membrane phospholipid turnover (32). GPE is associated with the progression from insulin sensitivity to insulin resistance (50, 51). Furthermore, serum GPE levels are increased in type 2 DM even in the absence of obesity (50). In this study, elevated GPE levels were observed in both the plasma and the kidneys of HFD/STZ mice, which is consistent with a previous report (50). The ratio of GPE to phospholipid metabolites is a potential biomarker for metabolic stress (31, 33). For instance, the PE/GPE ratio is decreased in cancer and is associated with mitochondrial dysfunction, a key event in the development of diabetic complications (20, 21, 31). We found that the LPE/GPE ratio was negatively correlated with indicators of DM, including inflammation, glucose intolerance, and renal dysfunction, in both plasma and the kidney. Collectively, both a decrease in LPE and an increase in GPE may contribute to the progression of DM. Moreover, the LPE/GPE ratio in the plasma of HFD/STZ mice behaved similarly to that in the kidney and liver, indicating that the plasma LPE/GPE ratio could reflect the disruption of PE metabolism in the metabolic organs under diabetic status.

Our LC-MS/MS analysis demonstrated an association between alterations in PEs and those in LPEs. Plasma PEs primarily containing FA 18:0 and 20:4 as acyl chains exhibited an increase in both HFD and HFD/STZ groups compared with the control, which might be associated with the increase in plasma LPE 18:0 and 20:4, their catabolites. In contrast, plasma PEs containing FA 16:0, 18:2, and 20:5 as acyl chains were decreased in both HFD and HFD/STZ groups, which might be associated with the reduction in plasma LPE 16:0, 18:2, and 20:5. Further investigation is needed into the mechanisms underlying alterations in plasma PE and LPE. In the kidney, LPE 18:1 and 20:4, contrary to the other LPE species, were increased in the HFD/STZ group, which is suggested to be mainly associated with the increase in their precursor, PEs containing FA 18:1 and 20:4. LPCAT3 preferentially incorporates C 20:4 as an acyl chain into LPE, resulting in the production of PEs containing FA 20:4 (34). Therefore, the upregulation of *Lpcat3* gene expression in the kidneys of HFD/STZ mice may contribute to the elevated levels of PE containing FA 20:4 and, consequently, to the increase in LPE 20:4.

This study has several limitations. First, the utility of the LPE/GPE ratio in clinical samples has not been confirmed. Although the renal LPE/GPE ratio could differentiate between the control and HFD/STZ groups, it also demonstrated the capability to distinguish the HFD group from the healthy control group. To eliminate the possibility that the reduction in the LPE/GPE ratio is solely indicative of obesity rather than a change specific to DM, further studies utilizing alternative diabetic models, clinical samples, and evaluation using insulin levels are required. Second, the direct association between LPE reduction and DM pathology, especially systemic inflammation, has not been established. Further investigations, such as LPE administration in disease model mice and evaluation using more expansive inflammatory panels and transcriptomics within the tissue, are warranted. Moreover, the causal relationship between decreased LPE and inflammation in HFD/STZ mice has not fully excluded the possibility of inflammation induced solely by STZ administration, and therefore, further investigation is required. Third, the mechanisms underlying alteration in plasma PE metabolism have not been revealed. Finally, a discrepancy between the protein and gene levels was observed in the expression of PNPLA6 in the kidneys of HFD/STZ mice. Moreover, this study provides only associative evidence regarding the relationship between alterations in PNPLA6 expression and those in PE metabolites. The mechanism behind this needs to be clarified in the future.

## 5 Conclusion

In conclusion, we found the upregulation of PNPLA6 was associated with the dysregulation of PE metabolism, especially LPE degradation to GPE in HFD/STZ mice. The decrease in the LPE/GPE ratio could reflect the diabetic status, which suggests that both LPE reduction and GPE elevation contribute to the progression of DM. This study provides a new perspective on DM pathology to understand the onset and progression of DM and its complications.

## Data Availability

The original contributions presented in the study are included in the article/Supplementary material, further inquiries can be directed to the corresponding author.

## Footnotes

1
https://www.metaboanalyst.ca/MetaboAnalyst/

## Glossary

ALT: alanine aminotransferase
AST: aspartate aminotransferase
AUC: area under the curve
CI: confidence interval
CRE: creatinine
DM: diabetes mellitus
FA: fatty acid
FBG: fasting blood glucose
GPC: glycerophosphorylcholine
GPE: glycerophosphorylethanolamine
HFD: high-fat diet
IS: internal standard
LC-MS/MS: liquid chromatography-tandem mass spectrometry
LPCAT3/4: lysophosphatidylcholine acyltransferase 3/4
LPC: lysophosphatidylcholine
LPE: lysophosphatidylethanolamine
LPEAT1: lysophosphatidylethanolamine acyltransferase 1
MeOH: methanol
PC: phosphatidylcholine
PE: phosphatidylethanolamine
PLA: phospholipases A
PNPLA6–9: patatin like phospholipase domain containing 6–9
STZ: streptozotocin
UACR: urine albumin-to-creatinine ratio
