# Supplementary material for: Dysregulation of phosphatidylethanolamine metabolism associated with upregulation of PNPLA6 in high-fat diet/streptozotocin mice
Source: Front Nutr. 2026 Jul 7;13:1799991. doi: 10.3389/fnut.2026.1799991 (PMC13385503; doi:10.3389/fnut.2026.1799991)
Supplement: Supplementary file 1 [file Supplementary_file_1.docx]

Supplementary Material

# Supplementary Table 1. Optimized single reaction monitoring for LPEs.

|  | | | | | |
| --- | --- | --- | --- | --- | --- |
| Species | Ionization | Parent ion | Product ion | Collision energy | Tube lens |
|  |  | (*m/z*) | (*m/z*) | (V) | (V) |
| LPE 18:1(d7) | [M−H]^−^ | 485.3 | 288.2 | 35 | 117 |
| LPE 16:0 | [M−H]^−^ | 452.3 | 255.3 | 35 | 106 |
| LPE 18:0 | [M−H]^−^ | 480.3 | 283.3 | 31 | 118 |
| LPE 18:1 | [M−H]^−^ | 478.3 | 281.3 | 31 | 124 |
| LPE 18:2 | [M−H]^−^ | 476.3 | 279.3 | 30 | 92 |
| LPE 20:4 | [M−H]^−^ | 500.3 | 303.5 | 27 | 108 |
| LPE 20:5 | [M−H]^−^ | 498.3 | 301.2 | 25 | 110 |
| LPE 22:6 | [M−H]^−^ | 524.3 | 327.2 | 23 | 111 |

LPE: lysophosphatidylethanolamine.

**Supplementary Table 2. Results of recovery and reproducibility for the extraction (n = 4).**

|  | | | | | | | |  |  |
| --- | --- | --- | --- | --- | --- | --- | --- | --- | --- |
|  |  | Kidney | | | Liver | | | | |
|  |  | Recovery | Intraday | Interday | Recovery | Intraday | Interday | |  |
| LPE species | | Mean ± SD (%) | CV (%) | CV (%) | Mean ± SD (%) | CV (%) | CV (%) | |  |
| LPE 16:0 | High | 84.8 ± 3.5 | 4.2 | 2.6 | 92.8 ± 1.5 | 1.6 | 2.2 | |  |
|  | Medium | 86.8 ± 1.3 | 1.5 | 2.9 | 89.1 ± 2.5 | 2.8 | 4.4 | |  |
|  | Low | 89.3 ± 4.0 | 4.4 | 0.7 | 83.6 ± 4.3 | 5.1 | 1.7 | |  |
| LPE 18:0 | High | 83.2 ± 5.2 | 6.3 | 1.4 | 93.8 ± 2.4 | 2.5 | 1.3 | |  |
|  | Medium | 88.2 ± 0.9 | 1.1 | 2.2 | 89.5 ± 1.8 | 2.0 | 2.0 | |  |
|  | Low | 84.8 ± 3.8 | 4.5 | 2.7 | 84.7 ± 2.6 | 3.1 | 2.5 | |  |
| LPE 18:1 | High | 87.7 ± 1.5 | 1.7 | 2.2 | 93.4 ± 2.3 | 2.4 | 1.7 | |  |
|  | Medium | 89.1 ± 1.4 | 1.6 | 3.1 | 87.5 ± 1.2 | 1.4 | 2.2 | |  |
|  | Low | 92.4 ± 4.6 | 4.9 | 1.8 | 85.2 ± 2.0 | 2.4 | 1.4 | |  |
| LPE 18:2 | High | 86.7 ± 3.1 | 3.5 | 3.0 | 90.9 ± 2.7 | 3.0 | 1.8 | |  |
|  | Medium | 90.7 ± 0.8 | 0.9 | 2.5 | 87.2 ± 2.2 | 2.5 | 2.8 | |  |
|  | Low | 92.7 ± 4.7 | 5.1 | 1.3 | 86.8 ± 2.7 | 3.1 | 2.4 | |  |
| LPE 20:4 | High | 88.3 ± 2.6 | 3.0 | 2.8 | 92.9 ± 5.4 | 5.8 | 2.2 | |  |
|  | Medium | 87.5 ± 0.7 | 0.8 | 5.5 | 87.5 ± 1.8 | 2.0 | 5.5 | |  |
|  | Low | 89.8 ± 5.9 | 6.5 | 9.2 | 85.5 ± 3.1 | 3.6 | 3.6 | |  |
| LPE 20:5 | High | 103.0 ± 4.0 | 3.9 | 17.4 | 91.1 ± 6.2 | 6.8 | 10.5 | |  |
|  | Medium | 98.4 ± 6.0 | 6.1 | 12.9 | 97.8 ± 22.7 | 23.2 | 17.6 | |  |
|  | Low | 80.6 ± 6.9 | 8.5 | 20.4 | 100.6 ± 14.2 | 14.2 | 13.7 | |  |
| LPE 22:6 | High | 87.1 ± 2.5 | 2.9 | 2.4 | 94.2 ± 2.5 | 2.7 | 1.9 | |  |
|  | Medium | 94.7 ± 4.9 | 5.2 | 6.4 | 90.8 ± 2.7 | 3.0 | 2.9 | |  |
|  | Low | 95.0 ± 4.2 | 4.4 | 3.6 | 85.2 ± 2.8 | 3.3 | 1.4 | |  |

Results are expressed as mean ± SD. The recovery rates were confirmed by spiking known concentrations of LPE standards (Low: 25 pmol; Medium: 50 pmol; High: 100 pmol) to homogenized murine organ samples (5 mg). CV: coefficient of variation; LPE: lysophosphatidylethanolamine; SD: standard deviation.

**Supplementary Table 3. Determination of linearity, LOD, and LOQ of LPEs.**

|  | | | | | |
| --- | --- | --- | --- | --- | --- |
| Species | Linearity | R^2^ | Range | LOD | LOQ |
|  |  |  | (fmol) | (fmol) | (fmol) |
| LPE 16:0 | y = 1.0282x | 0.9987 | 3.1–100000 | 3.1 | 6.1 |
| LPE 18:0 | y = 1.6278x | 0.9994 | 12.2–100000 | 12.2 | 24.4 |
| LPE 18:1 | y = 1.7822x | 0.9993 | 3.1–100000 | 3.1 | 6.1 |
| LPE 18:2 | y = 0.9368x | 0.9988 | 3.1–100000 | 3.1 | 6.1 |
| LPE 20:4 | y = 0.0818x | 0.9989 | 195.3–100000 | 195.3 | 390.6 |
| LPE 20:5 | y = 0.0726x | 0.9943 | 390.6–100000 | 390.6 | 781.3 |
| LPE 22:6 | y = 0.2308x | 0.9996 | 48.8–100000 | 48.8 | 97.7 |

A series of diluted LPE standard solutions at final concentrations of 0.00061, 0.00122, 0.00244, 0.00488, 0.00977, 0.01953, 0.03906, 0.07813, 0.15625, 0.31250, 0.625, 1.25, 2.5, 5.0, 10, 20 nmol/mL with a constant amount of internal standard (final concentration: 0.21 μmol/L) was injected to evaluate the linearity. The injection volume of the LC-MS/MS system was 5 μL. The LOD and LOQ were evaluated at signal-to-noise ratios of 3 and 10, respectively. LC-MS/MS: liquid chromatography-tandem mass spectrometry; LOD: limit of detection; LOQ: limit of quantification; LPE: lysophosphatidylethanolamine.

**Supplementary Table 4. Primer sequences of each gene used for qPCR.**

|  | | |
| --- | --- | --- |
| Gene | Species | Primer sequence (5'–3') |
| *β-actin* | Mouse | F: ACCACACCTTCTACAATGAG |
|  |  | R: ACGACCAGAGGCATACAG |
| *Pnpla6* | Mouse | F: AAGCCGGCACCATCATAG |
|  |  | R: TCAATCATGCGCTGGTACAC |
| *Pnpla7* | Mouse | F: GGAGGGGGTGGAGCTAGA |
|  |  | R: CGCCACACTCTGCTAGTGC |
| *Pnpla8* | Mouse | F: CCAAGCTCTCTAATGTTATCAGCA |
|  |  | R: AAGGCAACAGGCCATCAA |
| *Pnpla9* | Mouse | F: TCAGGATCTCATG CCCATCTCT |
|  |  | R: TGGTCGTGACTCCGCTTCTC |
| *Lpcat3* | Mouse | F: TCAGGATACCTGATTTGCTTCCA |
|  |  | R: GGATGGTCTGTTGCACCAAGTAG |
| *Lpcat4* | Mouse | F: TTCGGTTTCAGAGGATACGACAA |
|  |  | R: AATGTCTGGATTGTCGGACTGAA |
| *Lpeat1* | Mouse | F: CTGAAATGTGTGTGCTATGAGCG |
|  |  | R: TGGAAGAGAGGAAGTGGTGTCTG |

Lpcat: lysophosphatidylcholine acyltransferase; Lpeat: lysophosphatidylethanolamine acyltransferase; Pnpla: patatin like phospholipase domain containing; qPCR: quantitative polymerase chain reaction.

**Supplementary Table 5. Results of biological indices in each group.**

|  | | | |
| --- | --- | --- | --- |
|  | Control | HFD | HFD/STZ |
| FBG (mg/dL) | 72.8 ± 11.4^a^ | 89.0 ± 7.6^ab^ | 246.0 ± 47.3^b^ |
| CRE (mg/dL) | 0.13 ± 0.05^a^ | 0.14 ± 0.09^a^ | 0.37 ± 0.04^b^ |
| UACR (μg/mgCre) | 17.2 ± 3.7^a^ | 39.8 ± 4.6^b^ | 132.8 ± 82.9^b^ |
| AST (U/L) | 52.8 ± 17.5^a^ | 109.7 ± 30.4^b^ | 113.4 ± 14.2^b^ |
| ALT (U/L) | 24.1 ± 6.9^a^ | 80.2 ± 26.4^b^ | 62.8 ± 9.5^ab^ |

Different letters indicate statistical significance. Results are expressed as mean ± standard deviation. ALT: alanine aminotransferase; AST: aspartate aminotransferase; CRE: creatinine; FBG: fasting blood glucose; HFD: high-fat diet; STZ: streptozotocin; UACR: urine albumin-to-creatinine ratio.

**Supplementary Table 6. The detailed absolute LPE levels in the plasma samples.**

|  | | | | |
| --- | --- | --- | --- | --- |
|  | Species | Control | HFD | HFD/STZ |
|  |  | (nmol/mL) | (nmol/mL) | (nmol/mL) |
| Plasma | LPE 16:0 | 2.92 ± 0.25 | 2.78 ± 0.35 | 2.11 ± 0.14 |
|  | LPE 18:0 | 2.51 ± 0.22 | 4.08 ± 0.34 | 3.76 ± 0.38 |
|  | LPE 18:1 | 1.28 ± 0.22 | 2.17 ± 0.38 | 1.73 ± 0.26 |
|  | LPE 18:2 | 5.85 ± 0.43 | 1.46 ± 0.21 | 1.33 ± 0.26 |
|  | LPE 20:4 | 14.08 ± 1.48 | 42.97 ± 4.18 | 39.51 ± 4.74 |
|  | LPE 20:5 | 2.08 ± 0.29 | N.D. | N.D. |
|  | LPE 22:6 | 10.87 ± 1.28 | 10.11 ± 0.91 | 9.79 ± 0.61 |
|  | Total LPE | 39.59 ± 3.47 | 63.57 ± 5.65 | 58.23 ± 5.62 |

Results are expressed as mean ± standard deviation. HFD: high-fat diet; LPE: lysophosphatidylethanolamine; N.D., not detected; STZ: streptozotocin.

**Supplementary Table 7. The detailed absolute LPE levels in organ samples.**

|  | | | | |
| --- | --- | --- | --- | --- |
| Organs | Species | Control | HFD | HFD/STZ |
|  |  | (pmol/mg) | (pmol/mg) | (pmol/mg) |
| Kidney | LPE 16:0 | 21.46 ± 5.12 | 10.53 ± 1.54 | 9.78 ± 0.55 |
|  | LPE 18:0 | 46.80 ± 10.42 | 28.78 ± 5.39 | 29.29 ± 2.42 |
|  | LPE 18:1 | 12.74 ± 1.52 | 16.32 ± 2.26 | 16.27 ± 0.87 |
|  | LPE 18:2 | 4.96 ± 0.52 | 1.44 ± 0.11 | 1.22 ± 0.24 |
|  | LPE 20:4 | 126.90 ± 16.46 | 145.77 ± 17.42 | 150.57 ± 9.76 |
|  | LPE 20:5 | 11.08 ± 2.08 | N.D. | N.D. |
|  | LPE 22:6 | 35.53 ± 2.23 | 18.91 ± 1.41 | 17.83 ± 1.52 |
|  | Total LPE | 259.47 ± 34.09 | 221.75 ± 25.96 | 224.95 ± 8.68 |
|  |  |  |  |  |
| Liver | LPE 16:0 | 53.81 ± 14.31 | 23.81 ± 3.60 | 20.96 ± 3.56 |
|  | LPE 18:0 | 51.06 ± 16.98 | 33.21 ± 5.22 | 36.49 ± 6.01 |
|  | LPE 18:1 | 21.24 ± 4.57 | 24.87 ± 4.21 | 22.79 ± 3.64 |
|  | LPE 18:2 | 12.16 ± 3.77 | 2.39 ± 0.47 | 2.33 ± 0.56 |
|  | LPE 20:4 | 111.18 ± 35.82 | 187.61 ± 34.21 | 183.47 ± 66.47 |
|  | LPE 20:5 | 13.98 ± 5.68 | N.D. | N.D. |
|  | LPE 22:6 | 26.35 ± 10.26 | 18.23 ± 3.36 | 18.01 ± 2.93 |
|  | Total LPE | 289.79 ± 89.23 | 290.13 ± 44.93 | 284.05 ± 31.87 |

Results are expressed as mean ± standard deviation. HFD: high-fat diet; LPE: lysophosphatidylethanolamine; N.D.: not detected; STZ: streptozotocin.

**Supplementary Table 8. Results of the receiver operating characteristic curve analysis of LPE species.**

|  | | | | |
| --- | --- | --- | --- | --- |
|  |  |  | AUC (95% CI) |  |
|  |  | control vs HFD | control vs HFD/STZ | HFD vs HFD/STZ |
| Plasma | LPE 16:0 | 0.578 (0.2755–0.8807) | 1.000** (1.000–1.000) | 1.000** (1.000–1.000) |
|  | LPE 18:0 | 1.000*** (1.000–1.000) | 1.000** (1.000–1.000) | 0.750 (0.481–1.000) |
|  | LPE 18:1 | 0.984** (0.936–1.000) | 0.917** (0.767–1.000) | 0.833* (0.6048–1.000) |
|  | LPE 18:2 | 1.000*** (1.000–1.000) | 1.000** (1.000–1.000) | 0.646 (0.337–0.955) |
|  | LPE 20:4 | 1.000*** (1.000–1.000) | 1.000** (1.000–1.000) | 0.667 (0.370–0.964) |
|  | LPE 20:5 | 1.000*** (1.000–1.000) | 1.000** (1.000–1.000) | 0.500 (0.184–0.816) |
|  | LPE 22:6 | 0.594 (0.304–0.884) | 0.771 (0.519–1.000) | 0.667 (0.372–0.962) |
|  |  |  |  |  |
| Kidney | LPE 16:0 | 1.000*** (1.000–1.000) | 1.000** (1.000–1.000) | 0.604 (0.292–0.916) |
|  | LPE 18:0 | 0.969** (0.892–1.000) | 1.000** (1.000–1.000) | 0.625 (0.304–0.946) |
|  | LPE 18:1 | 0.938** (0.809–1.000) | 0.979** (0.915–1.000) | 0.583 (0.263–0.904) |
|  | LPE 18:2 | 1.000*** (1.000–1.000) | 1.000** (1.000–1.000) | 0.833* (0.535–1.000) |
|  | LPE 20:4 | 0.875* (0.698–1.000) | 0.875* (0.646–1.000) | 0.625 (0.290–0.961) |
|  | LPE 20:5 | 1.000*** (1.000–1.000) | 1.000** (1.000–1.000) | 0.500 (0.184–0.816) |
|  | LPE 22:6 | 1.000*** (1.000–1.000) | 1.000** (1.000–1.000) | 0.646 (0.338–0.954) |
|  |  |  |  |  |
| Liver | LPE 16:0 | 1.000*** (1.000–1.000) | 1.000** (1.000–1.000) | 0.729 (0.451–1.000) |
|  | LPE 18:0 | 0.938** (0.822–1.000) | 0.816 (0.576–1.000) | 0.708 (0.425–0.992) |
|  | LPE 18:1 | 0.766 (0.525–1.000) | 0.646 (0.334–0.958) | 0.563 (0.236–0.889) |
|  | LPE 18:2 | 1.000*** (1.000–1.000) | 1.000** (1.000–1.000) | 0.542 (0.214–0.869) |
|  | LPE 20:4 | 0.938** (0.809–1.000) | 0.896* (0.698–1.000) | 0.521 (0.195–0.847) |
|  | LPE 20:5 | 1.000*** (1.000–1.000) | 1.000** (1.000–1.000) | 0.500 (0.184–0.816) |
|  | LPE 22:6 | 0.828* (0.617–1.000) | 0.854* (0.652–1.000) | 0.521 (0.192–0.850) |

**p* < 0.05, ***p* < 0.01, ****p* < 0.001. AUC: area under the curve; CI: confidence interval; GPE: glycerophosphorylethanolamine; HFD: high-fat diet; LPE: lysophosphatidylethanolamine; STZ: streptozotocin.

**Supplementary Table 9. Correlation analysis between LPE species and biological indices.**

|  | | | | | | |
| --- | --- | --- | --- | --- | --- | --- |
|  |  | AST | ALT | FBG | CRE | UACR |
| Plasma | LPE 16:0 | −0.468 | −0.196 | −0.890**** | −0.645* | −0.763*** |
|  | LPE 18:0 | 0.764** | 0.764** | 0.375 | 0.396 | 0.537* |
|  | LPE 18:1 | 0.561* | 0.750** | 0.266 | 0.123 | 0.314 |
|  | LPE 18:2 | −0.621* | −0.521* | −0.592* | −0.407 | −0.849**** |
|  | LPE 20:4 | 0.714** | 0.825*** | 0.467 | 0.438 | 0.521* |
|  | LPE 20:5 | −0.793*** | −0.789*** | −0.640* | −0.500 | −0.797**** |
|  | LPE 22:6 | −0.182 | 0.004 | −0.256 | −0.172 | −0.370 |
|  |  |  |  |  |  |  |
| Kidney | LPE 16:0 | −0.764** | −0.846*** | −0.610* | −0.546* | −0.693** |
|  | LPE 18:0 | −0.768** | −0.879**** | −0.443 | −0.429 | −0.558* |
|  | LPE 18:1 | 0.332 | 0.275 | 0.660** | 0.484 | 0.760*** |
|  | LPE 18:2 | −0.679** | −0.686** | −0.627* | −0.594* | −0.783**** |
|  | LPE 20:4 | 0.114 | 0.057 | 0.497 | 0.383 | 0.600** |
|  | LPE 20:5 | −0.827**** | −0.823*** | −0.667** | −0.469 | −0.780**** |
|  | LPE 22:6 | −0.696** | −0.636* | −0.629* | −0.585* | −0.781**** |
|  |  |  |  |  |  |  |
| Liver | LPE 16:0 | −0.539* | −0.586* | −0.534* | −0.403 | −0.770*** |
|  | LPE 18:0 | −0.386 | −0.546* | −0.0215 | 0.024 | −0.458* |
|  | LPE 18:1 | 0.236 | 0.296 | 0.567* | 0.066 | 0.144 |
|  | LPE 18:2 | −0.671** | −0.671** | −0.390 | −0.469 | −0.698*** |
|  | LPE 20:4 | 0.521* | 0.571* | 0.697** | 0.304 | 0.490* |
|  | LPE 20:5 | −0.806*** | −0.802*** | −0.631* | −0.515 | −0.768*** |
|  | LPE 22:6 | −0.471 | −0.382 | 0.061 | −0.240 | −0.521* |

**p* < 0.05, ***p* < 0.01, ****p* < 0.001, *****p* < 0.0001. ALT: alanine aminotransferase; AST: aspartate aminotransferase; CRE: creatinine; FBG: fasting blood glucose; GPE: glycerophosphorylethanolamine; HFD: high-fat diet; LPE: lysophosphatidylethanolamine; STZ: streptozotocin; UACR: urine albumin-to-creatinine ratio.

**Supplementary Table 10. The detailed levels of PE metabolites in the plasma samples.**

|  | | | |
| --- | --- | --- | --- |
|  | Control | HFD | HFD/STZ |
| Species | (nmol/mL) | (nmol/mL) | (nmol/mL) |
| PE (16:0/18:1) | 1.69 ± 0.40 | 1.40 ± 0.27 | 1.45 ± 0.18 |
| PE (16:0/18:2) | 2.96 ± 0.51 | 0.47 ± 0.11 | 0.52 ± 0.08 |
| PE (18:1/18:2) | 1.79 ± 0.43 | 0.66 ± 0.14 | 0.84 ± 0.15 |
| PE (16:0/20:4) | 2.24 ± 0.47 | 4.40 ± 0.81 | 5.12 ± 0.58 |
| PE (18:0/20:3) | 0.48 ± 0.10 | 1.47 ± 0.25 | 1.99 ± 0.27 |
| PE (18:0/20:4) | 5.01 ± 0.93 | 13.12 ± 2.26 | 17.64 ± 2.35 |
| PE (18:1/20:4) | 3.79 ± 0.94 | 10.74 ± 2.18 | 11.27 ± 1.48 |
| PE (16:0/22:6) | 10.12 ± 1.95 | 8.26 ± 1.64 | 10.76 ± 1.04 |
| PE (18:2/20:5) | 0.24 ± 0.05 | 0.18 ± 0.04 | 0.22 ± 0.03 |
| PE (20:0/20:4) | 0.23 ± 0.03 | 0.92 ± 0.08 | 1.00 ± 0.19 |
| PE (18:0/22:5) | 0.28 ± 0.06 | 0.86 ± 0.23 | 0.87 ± 0.15 |
| PE (18:0/22:6) | 4.51 ± 0.70 | 5.20 ± 0.90 | 6.59 ± 0.76 |
| PE (18:1/22:6) | 2.40 ± 0.54 | 3.11 ± 0.61 | 4.05 ± 0.48 |
| total PE | 35.74 ± 6.70 | 50.79 ± 9.01 | 62.32 ± 7.03 |
| GPE | 0.012 ± 0.004 | 0.022 ± 0.007 | 0.030 ± 0.008 |

Results are expressed as mean ± standard deviation. GPE: glycerophosphorylethanolamine; HFD: high-fat diet; PE: phosphatidylethanolamine; STZ: streptozotocin.

**Supplementary Table 11. The detailed levels of PE metabolites in the kidney samples.**

|  | | | |
| --- | --- | --- | --- |
|  | Control | HFD | HFD/STZ |
| Species | (pmol/mg) | (pmol/mg) | (pmol/mg) |
| PE (16:0/18:1) | 519.55 ± 103.10 | 577.75 ± 92.90 | 767.22 ± 436.16 |
| PE (16:0/18:2) | 261.22 ± 34.96 | 67.59 ± 12.36 | 69.35 ± 34.15 |
| PE (18:1/18:2) | 316.11 ± 57.37 | 170.09 ± 37.49 | 206.37 ± 109.24 |
| PE (16:0/20:4) | 1478.67 ± 219.19 | 1744.80 ± 306.73 | 2280.95 ± 1185.38 |
| PE (18:0/20:3) | 864.46 ± 129.75 | 1172.76 ± 204.47 | 1612.29 ± 916.84 |
| PE (18:0/20:4) | 8029.36 ± 992.31 | 10272.49 ± 1813.85 | 14051.08 ± 7963.25 |
| PE (18:1/20:4) | 3616.97 ± 634.32 | 4400.55 ± 1152.20 | 5803.70 ± 3387.04 |
| PE (16:0/22:6) | 1618.61 ± 223.07 | 800.10 ± 94.08 | 1071.84 ± 583.86 |
| PE (18:2/20:5) | 580.95 ± 227.35 | 131.90 ± 15.15 | 102.98 ± 57.04 |
| PE (20:0/20:4) | 274.23 ± 49.05 | 493.47 ± 94.66 | 655.42 ± 353.35 |
| PE (18:0/22:5) | 180.68 ± 35.14 | 699.14 ± 205.68 | 599.78 ± 337.23 |
| PE (18:0/22:6) | 2161.15 ± 362.69 | 1889.54 ± 449.02 | 1926.64 ± 1154.83 |
| PE (18:1/22:6) | 885.34 ± 151.88 | 1064.21 ± 199.18 | 1454.15 ± 800.87 |
| total PE | 20787.27 ± 2827.15 | 23484.39 ± 4602.68 | 30601.78 ± 17263.90 |
| GPE | 3.39 ± 0.96 | 4.26 ± 1.20 | 6.31 ± 1.68 |

Results are expressed as mean ± standard deviation. GPE: glycerophosphorylethanolamine; HFD: high-fat diet; PE: phosphatidylethanolamine; STZ: streptozotocin.

**Supplementary Table 12. The detailed levels of PE metabolites in the liver samples.**

|  | | | |
| --- | --- | --- | --- |
|  | Control | HFD | HFD/STZ |
| Species | (pmol/mg) | (pmol/mg) | (pmol/mg) |
| PE (16:0/18:1) | 2172.08 ± 698.51 | 1691.57 ± 557.52 | 1127.15 ± 413.89 |
| PE (16:0/18:2) | 2542.89 ± 694.14 | 315.83 ± 118.31 | 259.32 ± 60.88 |
| PE (18:1/18:2) | 2279.64 ± 590.57 | 679.17 ± 207.47 | 602.46 ± 176.86 |
| PE (16:0/20:4) | 2385.41 ± 672.24 | 2844.83 ± 962.73 | 2234.11 ± 345.43 |
| PE (18:0/20:3) | 1066.43 ± 341.25 | 1767.16 ± 681.90 | 1486.64 ± 398.67 |
| PE (18:0/20:4) | 12087.87 ± 3033.51 | 18159.37 ± 6734.58 | 16773.12 ± 3964.14 |
| PE (18:1/20:4) | 8992.36 ± 3275.69 | 13775.93 ± 5028.69 | 9435.63 ± 1962.96 |
| PE (16:0/22:6) | 10265.74 ± 3074.53 | 6548.88 ± 2246.29 | 6159.67 ± 1026.77 |
| PE (18:2/20:5) | 605.93 ± 224.46 | 643.22 ± 221.06 | 385.14 ± 99.56 |
| PE (20:0/20:4) | 1257.51 ± 434.98 | 981.02 ± 328.80 | 923.90 ± 213.02 |
| PE (18:0/22:5) | 744.04 ± 223.95 | 2860.23 ± 1297.60 | 1665.31 ± 341.33 |
| PE (18:0/22:6) | 6721.57 ± 2310.49 | 6934.98 ± 2678.32 | 5209.11 ± 886.97 |
| PE (18:1/22:6) | 3731.00 ± 1086.29 | 3653.01 ± 1076.44 | 3491.32 ± 597.30 |
| total PE | 54852.49 ± 16005.86 | 60855.18 ± 21738.24 | 49752.89 ± 10035.14 |
| GPE | 1.12 ± 0.24 | 1.29 ± 0.23 | 2.75 ± 1.00 |

Results are expressed as mean ± standard deviation. GPE: glycerophosphorylethanolamine; HFD: high-fat diet; PE: phosphatidylethanolamine; STZ: streptozotocin.

**Supplementary Table 13. Results of the receiver operating characteristic curve analysis of PC metabolites.**

|  | | | | |
| --- | --- | --- | --- | --- |
|  |  |  | AUC (95% CI) |  |
|  |  | control vs HFD | control vs HFD/STZ | HFD vs HFD/STZ |
| Plasma | PC | 1.000*** (1.000–1.000) | 1.000** (1.000–1.000) | 0.500 (0.174–0.826) |
|  | LPC | 1.000*** (1.000–1.000) | 1.000** (1.000–1.000) | 0.725 (0.432–1.000) |
|  | GPC | 0.906** (0.757–1.000) | 0.925* (0.769–1.000) | 0.625 (0.295–0.955) |
|  | PC/GPC | 0.719 (0.450–0.987) | 0.775 (0.516–1.000) | 0.600 (0.283–0.917) |
|  | LPC/GPC | 0.672 (0.383–0.961) | 0.725 (0.437–1.000) | 0.525 (0.188–0.862) |
|  | PC/LPC | 0.828* (0.625–1.000) | 0.675 (0.375–0.975) | 0.725 (0.437–1.000) |
|  |  |  |  |  |
| Kidney | PC | 0.906** (0.757–1.000) | 0.917** (0.767–1.000) | 0.563 (0.229–0.896) |
|  | LPC | 1.000*** (1.000–1.000) | 1.000** (1.000–1.000) | 0.688 (0.393–0.982) |
|  | GPC | 0.703 (0.433–0.973) | 0.896* (0.720–1.000) | 0.833* (0.605–1.000) |
|  | PC/GPC | 0.922** (0.768–1.000) | 1.000** (1.000–1.000) | 0.854* (0.652–1.000) |
|  | LPC/GPC | 0.563 (0.270–0.855) | 0.875* (0.685–1.000) | 0.896* (0.720–1.000) |
|  | PC/LPC | 0.984** (0.936–1.000) | 0.979** (0.915–1.000) | 0.500 (0.180–0.820) |
|  |  |  |  |  |
| Liver | PC | 0.906** (0.757–1.000) | 0.958** (0.858–1.000) | 0.667 (0.369–0.964) |
|  | LPC | 0.891** (0.708–1.000) | 0.875* (0.685–1.000) | 0.667 (0.351–0.983) |
|  | GPC | 1.000*** (1.000–1.000) | 1.000** (1.000–1.000) | 0.917** (0.768–1.000) |
|  | PC/GPC | 1.000*** (1.000–1.000) | 1.000** (1.000–1.000) | 0.896* (0.718–1.000) |
|  | LPC/GPC | 1.000*** (1.000–1.000) | 1.000** (1.000–1.000) | 0.958** (0.858–1.000) |
|  | PC/LPC | 1.000*** (1.000–1.000) | 1.000** (1.000–1.000) | 0.563 (0.245–0.880) |

**p* < 0.05, ***p* < 0.01, ****p* < 0.001. AUC: area under the curve; CI: confidence interval; GPC: glycerophosphorylethanolcholine; HFD: high-fat diet; LPC: lysophosphatidylcholine; PC: phosphatidylcholine; STZ: streptozotocin.

**Supplementary Table 14. Correlation analysis between PC metabolites and biological indices.**

|  | | | | | | |
| --- | --- | --- | --- | --- | --- | --- |
|  |  | AST | ALT | FBG | CRE | UACR |
| Plasma | PC | 0.763** | 0.846*** | 0.411 | 0.418 | 0.750*** |
|  | LPC | 0.701** | 0.789** | 0.394 | 0.335 | 0.600** |
|  | GPC | 0.640* | 0.565* | 0.304 | 0.231 | 0.455 |
|  | PC/GPC | −0.314 | −0.213 | −0.231 | −0.121 | −0.193 |
|  | LPC/GPC | −0.327 | −0.182 | −0.198 | −0.247 | −0.108 |
|  | PC/LPC | −0.442 | −0.367 | −0.253 | 0.005 | −0.276 |
|  |  |  |  |  |  |  |
| Kidney | PC | −0.782*** | −0.696** | −0.549* | −0.592* | −0.530* |
|  | LPC | 0.689** | 0.746** | 0.456 | 0.319 | 0.653** |
|  | GPC | 0.432 | 0.200 | 0.613* | 0.449 | 0.600** |
|  | PC/GPC | −0.750** | −0.557* | −0.751** | −0.708** | −0.760*** |
|  | LPC/GPC | −0.250 | −0.039 | −0.531* | −0.422 | −0.374 |
|  | PC/LPC | −0.829*** | −0.743** | −0.492 | −0.537 | −0.625** |
|  |  |  |  |  |  |  |
| Liver | PC | −0.514 | −0.496 | −0.658** | −0.387 | −0.628** |
|  | LPC | 0.504 | 0.600* | 0.608* | 0.266 | 0.375 |
|  | GPC | 0.646* | 0.536* | 0.851*** | 0.638* | 0.800**** |
|  | PC/GPC | −0.621* | −0.539* | −0.819*** | −0.543* | −0.819**** |
|  | LPC/GPC | −0.718** | −0.575* | −0.795*** | −0.726** | −0.893**** |
|  | PC/LPC | −0.532* | −0.529* | −0.693** | −0.319 | −0.672** |

**p* < 0.05, ***p* < 0.01, ****p* < 0.001, *****p* < 0.0001. ALT: alanine aminotransferase; AST: aspartate aminotransferase; CRE: creatinine; FBG: fasting blood glucose; GPC: glycerophosphorylethanolcholine; HFD: high-fat diet; LPC: lysophosphatidylcholine; PC: phosphatidylcholine; STZ: streptozotocin; UACR: urine albumin-to-creatinine ratio.

**Supplementary Table 15. The detailed levels of PC metabolites in the plasma samples.**

|  | | | |
| --- | --- | --- | --- |
|  | Control | HFD | HFD/STZ |
| Species | (nmol/mL) | (nmol/mL) | (nmol/mL) |
| PC (16:0/16:0) | 20.67 ± 4.14 | 26.06 ± 6.92 | 20.23 ± 2.18 |
| PC (16:0/18:1) | 283.18 ± 57.98 | 584.75 ± 146.51 | 544.53 ± 52.62 |
| PC (16:0/18:2) | 590.85 ± 96.03 | 261.03 ± 54.26 | 267.80 ± 21.33 |
| PC (18:0/18:1) | 91.27 ± 23.10 | 502.00 ± 167.66 | 476.41 ± 99.50 |
| PC (18:0/18:2) | 572.64 ± 119.96 | 371.47 ± 92.15 | 376.77 ± 40.69 |
| PC (18:1/18:2) | 135.73 ± 30.18 | 132.81 ± 35.20 | 135.24 ± 30.00 |
| PC (16:0/20:4) | 158.31 ± 33.52 | 596.53 ± 132.21 | 521.01 ± 69.64 |
| PC (16:1/20:4) | 84.78 ± 19.02 | 66.27 ± 21.19 | 55.82 ± 19.07 |
| PC (18:0/20:4) | 173.47 ± 40.76 | 1024.64 ± 272.81 | 951.06 ± 197.32 |
| PC (18:1/20:4) | 50.46 ± 10.21 | 244.79 ± 58.99 | 202.26 ± 31.67 |
| PC (16:0/22:6) | 202.44 **±** 34.46 | 287.24 ± 68.50 | 276.33 ± 32.02 |
| PC (18:2/22:6) | 10.17 ± 2.46 | 24.61 ± 7.55 | 18.27 ± 4.64 |
| total PC | 2373.98 ± 456.91 | 4122.19 ± 1049.68 | 3845.75 ± 525.99 |
| LPC 14:0 | 0.63 ± 0.12 | 0.94 ± 0.24 | 0.55 ± 0.07 |
| LPC 16:0 | 95.87 ± 12.47 | 153.09 ± 34.13 | 128.16 ± 10.32 |
| LPC 16:1 | 4.43 ± 0.81 | 7.35 ± 1.98 | 5.75 ± 0.92 |
| LPC 17:0 | 2.77 ± 0.44 | 8.20 ± 1.84 | 6.57 ± 0.89 |
| LPC 18:0 | 35.67 ± 4.56 | 106.58 ± 22.69 | 94.03 ± 10.40 |
| LPC 18:1 | 26.51 ± 5.17 | 85.47 ± 22.11 | 75.81 ± 8.80 |
| LPC 18:2 | 60.19 ± 10.42 | 44.43 ± 11.07 | 40.44 ± 4.22 |
| LPC 20:3 | 8.73 ± 2.01 | 21.54 ± 4.88 | 18.62 ± 2.21 |
| LPC 20:4 | 12.65 ± 2.90 | 82.12 ± 27.53 | 68.23 ± 13.31 |
| LPC 22:6 | 17.35 ± 3.32 | 29.58 ± 8.56 | 26.97 ± 7.07 |
| total LPC | 264.79 ± 40.28 | 539.30 ± 131.89 | 465.12 ± 53.09 |
| GPC | 3.67 ± 1.70 | 8.37 ± 3.36 | 7.42 ± 2.28 |

Results are expressed as mean ± standard deviation. GPC: glycerophosphorylcholine; HFD: high-fat diet; LPC: lysophosphatidylcholine; PC: phosphatidylcholine; STZ: streptozotocin.

**Supplementary Table 16. The detailed levels of PC metabolites in the kidney samples.**

|  | | | |
| --- | --- | --- | --- |
|  | Control | HFD | HFD/STZ |
| Species | (pmol/mg) | (pmol/mg) | (pmol/mg) |
| PC (16:0/16:0) | 4122.95 ± 2074.34 | 2099.12 ± 402.81 | 1805.12 ± 391.11 |
| PC (16:0/18:1) | 2832.35 ± 1335.17 | 1983.62 ± 399.93 | 1984.10 ± 411.66 |
| PC (16:0/18:2) | 4082.37 ± 2031.76 | 1242.52 ± 204.67 | 1022.61 ± 211.26 |
| PC (18:0/18:1) | 2447.61 ± 1392.46 | 2058.74 ± 330.56 | 2442.68 ± 1146.34 |
| PC (18:0/18:2) | 4673.9 ± 2273.34 | 1410.53 ± 292.64 | 1483.42 ± 407.81 |
| PC (18:1/18:2) | 1570.36 ± 756.33 | 510.37 ± 109.92 | 491.96 ± 72.19 |
| PC (16:0/20:4) | 3257.16 ± 1629.43 | 2863.30 ± 518.88 | 2306.72 ± 519.45 |
| PC (16:1/20:4) | 1994.11 ± 1013.67 | 392.98 ± 86.54 | 301.67 ± 85.24 |
| PC (18:0/20:4) | 2956.61 ± 1320.09 | 2862.96 ± 470.43 | 2913.53 ± 887.94 |
| PC (18:1/20:4) | 776.40 ± 309.68 | 1280.40 ± 212.11 | 1046.00 ± 198.28 |
| PC (16:0/22:6) | 6843.68 ± 3058.21 | 3661.03 ± 801.05 | 3401.04 ± 833.66 |
| PC (18:2/22:6) | 1719.88 ± 891.69 | 711.33 ± 162.31 | 653.55 ± 188.25 |
| total PC | 37277.39 ± 17905.77 | 21076.90 ± 3537.68 | 19852.39 ± 5056.42 |
| LPC 14:0 | 0.39 ± 0.06 | 0.46 ± 0.07 | 0.32 ± 0.02 |
| LPC 16:0 | 105.61 ± 7.10 | 110.19 ± 14.47 | 102.62 ± 6.48 |
| LPC 16:1 | 2.99 ± 0.54 | 4.01 ± 0.55 | 3.31 ± 0.33 |
| LPC 17:0 | 3.48 ± 0.30 | 6.50 ± 0.73 | 5.58 ± 0.35 |
| LPC 18:0 | 49.88 ± 5.17 | 87.32 ± 9.29 | 81.43 ± 5.31 |
| LPC 18:1 | 21.16 ± 1.63 | 42.44 ± 3.83 | 41.14 ± 2.60 |
| LPC 18:2 | 22.01 ± 2.70 | 10.37 ± 1.24 | 9.40 ± 0.81 |
| LPC 20:3 | 1.00 ± 0.10 | 2.41 ± 0.34 | 2.14 ± 0.32 |
| LPC 20:4 | 7.13 ± 0.74 | 21.24 ± 3.39 | 18.94 ± 1.66 |
| LPC 22:6 | 20.02 ± 1.83 | 14.80 ± 2.01 | 15.59 ± 0.90 |
| total LPC | 233.69 ± 18.31 | 299.74 ± 34.20 | 280.47 ± 15.81 |
| GPC | 28.26 ± 14.11 | 34.79 ± 14.51 | 52.76 ± 17.45 |

Results are expressed as mean ± standard deviation. GPC: glycerophosphorylcholine; HFD: high-fat diet; LPC: lysophosphatidylcholine; PC: phosphatidylcholine; STZ: streptozotocin.

**Supplementary Table 17. The detailed levels of PC metabolites in the liver samples.**

|  | | | |
| --- | --- | --- | --- |
|  | Control | HFD | HFD/STZ |
| Species | (pmol/mg) | (pmol/mg) | (pmol/mg) |
| PC (16:0/16:0) | 2104.30 ± 1101.38 | 511.34 ± 126.59 | 426.84 ± 96.59 |
| PC (16:0/18:1) | 16504.66 ± 8180.25 | 6161.56 ± 1956.80 | 5443.54 ± 1802.04 |
| PC (16:0/18:2) | 21778.13 ± 11758.48 | 2300.14 ± 680.69 | 2440.73 ± 910.79 |
| PC (18:0/18:1) | 3275.22 ± 1747.45 | 3052.20 ± 1038.60 | 2306.76 ± 617.31 |
| PC (18:0/18:2) | 20509.79 ± 11085.90 | 2702.12 ± 812.61 | 2480.63 ± 825.50 |
| PC (18:1/18:2) | 6654.01 ± 3312.63 | 1377.51 ± 416.15 | 1303.30 ± 479.14 |
| PC (16:0/20:4) | 9308.79 ± 4899.10 | 6383.14 ± 1855.00 | 5188.27 ± 1351.52 |
| PC (16:1/20:4) | 11296.02 ± 6319.54 | 1986.08 ± 627.00 | 1270.83 ± 388.82 |
| PC (18:0/20:4) | 10124.78 ± 5334.86 | 10158.37 ± 3085.37 | 7872.72 ± 2007.33 |
| PC (18:1/20:4) | 2903.54 ± 1407.82 | 2896.28 ± 890.06 | 2356.55 ± 582.76 |
| PC (16:0/22:6) | 10466.40 ± 5372.44 | 3562.15 ± 1089.22 | 3645.62 ± 751.33 |
| PC (18:2/22:6) | 1713.17 ± 942.61 | 571.67 ± 189.40 | 514.76 ± 130.47 |
| total PC | 116638.81 ± 608181.28 | 41662.56 ± 12149.32 | 35250.54 ± 9699.48 |
| LPC 14:0 | 0.70 ± 0.28 | 0.47 ± 0.08 | 0.32 ± 0.06 |
| LPC 16:0 | 132.99 ± 25.69 | 109.80 ± 12.42 | 105.50 ± 15.30 |
| LPC 16:1 | 10.84 ± 3.51 | 8.18 ± 1.61 | 6.13 ± 1.13 |
| LPC 17:0 | 8.65 ± 2.20 | 11.30 ± 2.10 | 10.94 ± 1.55 |
| LPC 18:0 | 58.94 ± 13.15 | 115.61 ± 17.17 | 112.61 ± 15.35 |
| LPC 18:1 | 33.85 ± 6.99 | 67.26 ± 11.75 | 60.99 ± 8.66 |
| LPC 18:2 | 42.00 ± 11.24 | 22.28 ± 4.86 | 20.31 ± 5.31 |
| LPC 20:3 | 1.63 ± 0.35 | 5.24 ± 0.84 | 4.93 ± 0.94 |
| LPC 20:4 | 13.53 ± 3.49 | 69.66 ± 13.70 | 54.89 ± 9.39 |
| LPC 22:6 | 18.73 ± 5.22 | 24.84 ± 6.33 | 23.52 ± 3.81 |
| total LPC | 321.89 ± 71.01 | 434.63 ± 60.34 | 400.13 ± 54.17 |
| GPC | 2.92 ± 1.10 | 9.72 ± 1.32 | 12.49 ± 1.75 |

Results are expressed as mean ± standard deviation. GPC: glycerophosphorylcholine; HFD: high-fat diet; LPC: lysophosphatidylcholine; PC: phosphatidylcholine; STZ: streptozotocin.


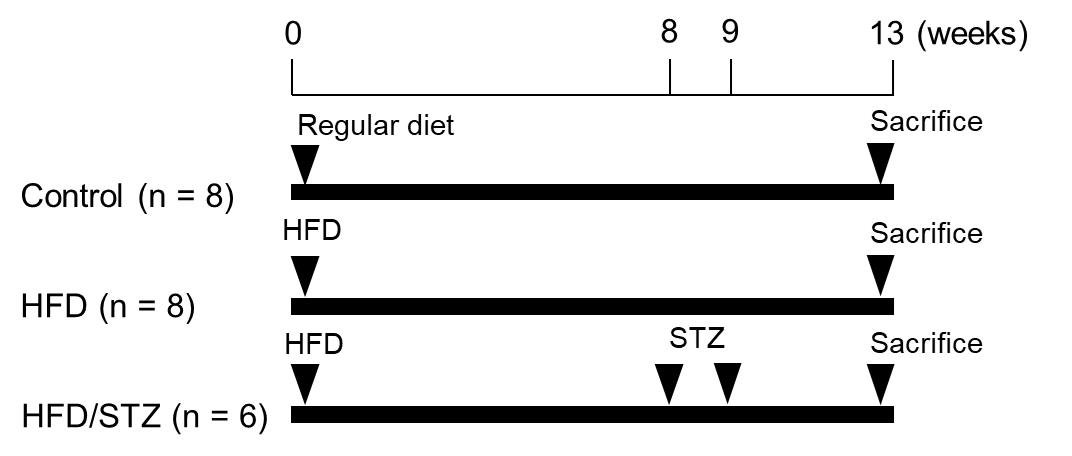
**Supplementary Figure 1.** Diagram of the experimental strategy. HFD: high-fat diet; STZ: streptozotocin.


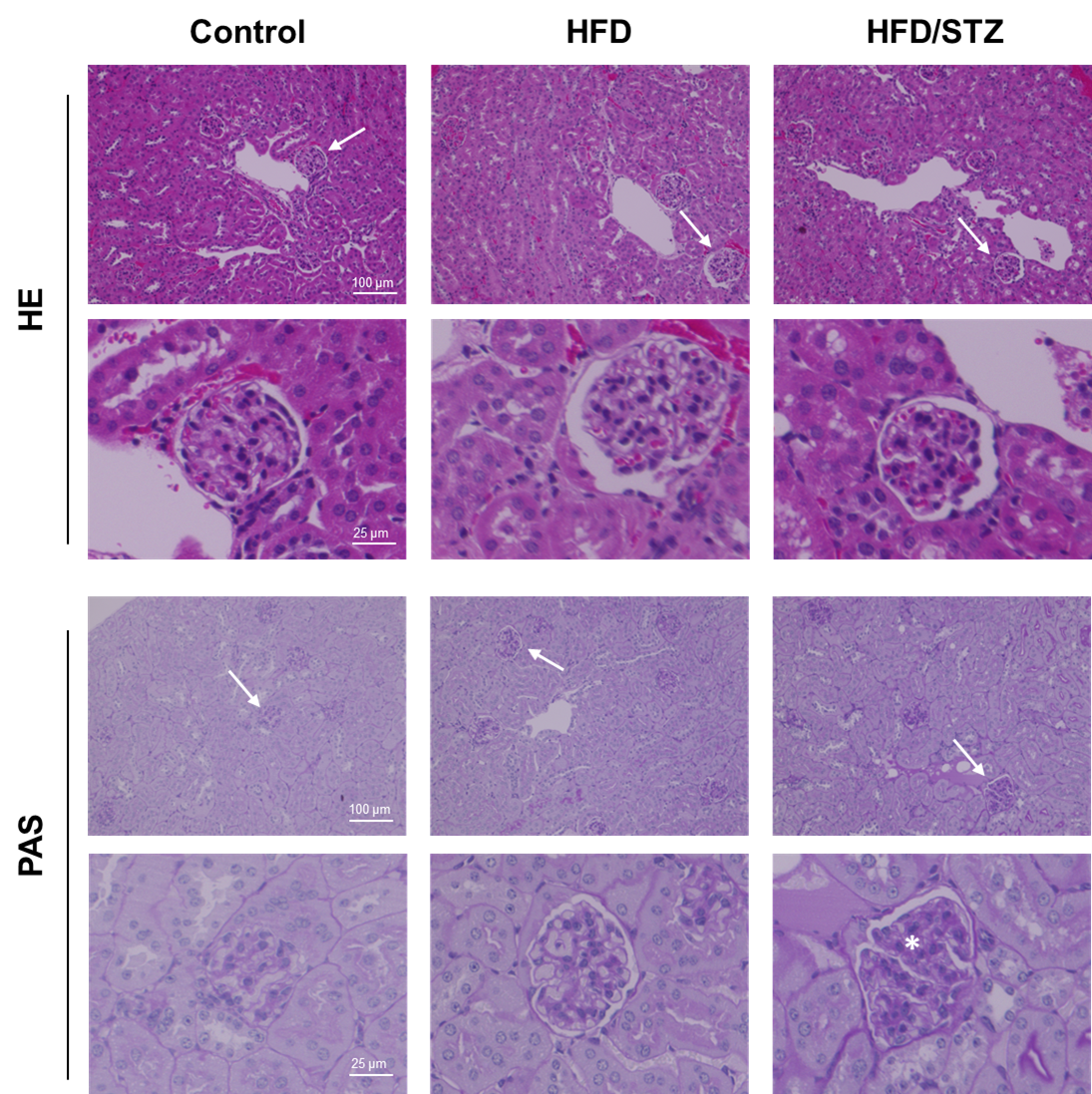
**Supplementary Figure 2.** Histology of the kidney samples. Arrows indicate representative glomeruli. PAS staining revealed mesangial expansion in the kidney of HFD/STZ mice (*). HE: Hematoxylin-Eosin; HFD: high-fat diet; PAS: Periodic acid-Schiff; STZ: streptozotocin.

**
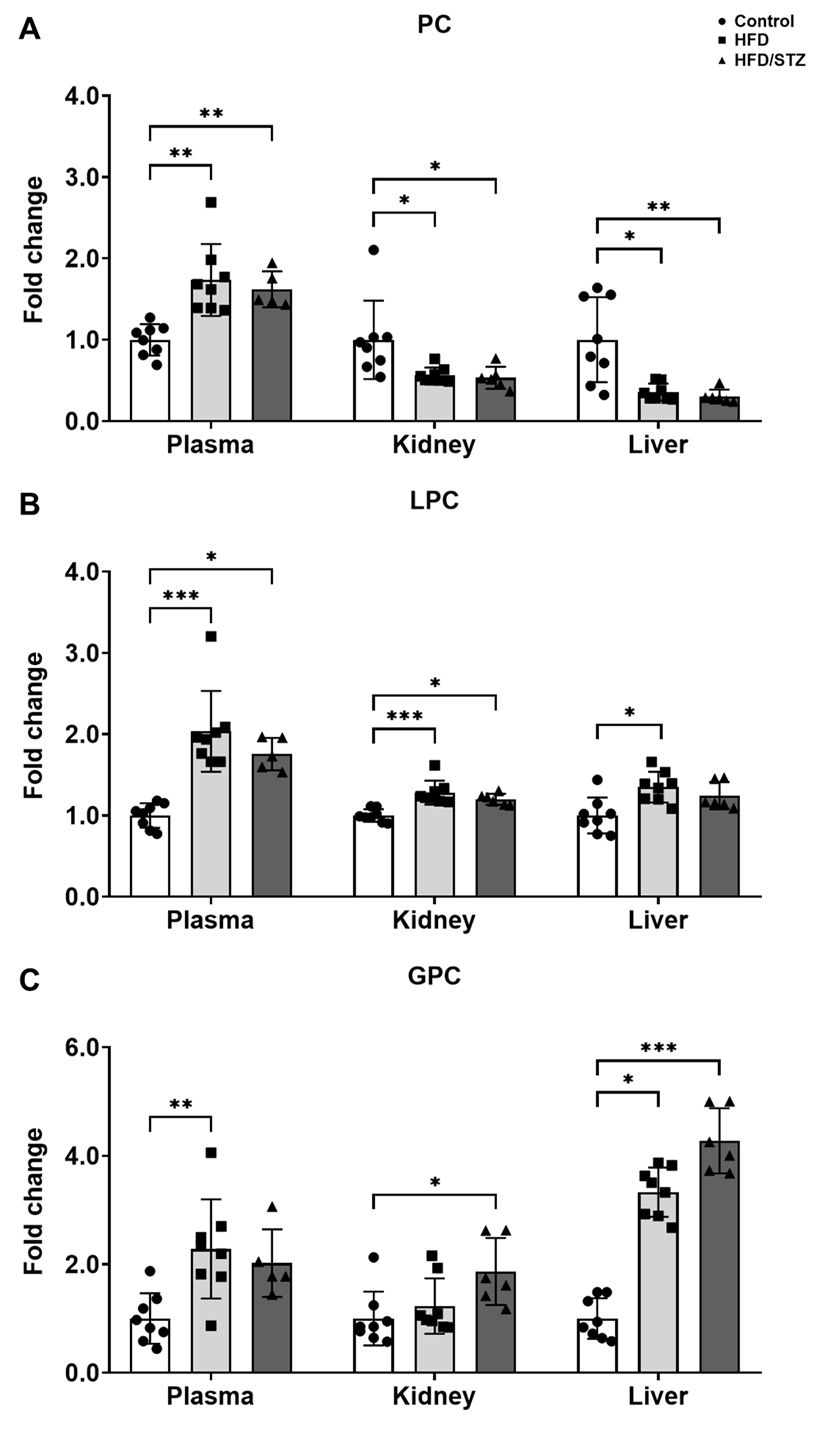
**

**Supplementary Figure 3**. Alterations in PC metabolites, including PC (A), LPC (B), and GPC (C), in the plasma, kidney, and liver samples (mean ± standard deviation). **p* < 0.05, ***p* < 0.01, ****p* < 0.001. GPC: glycerophosphorylcholine; HFD: high-fat diet; LPC: lysophosphatidylcholine; PC: phosphatidylcholine; STZ: streptozotocin.


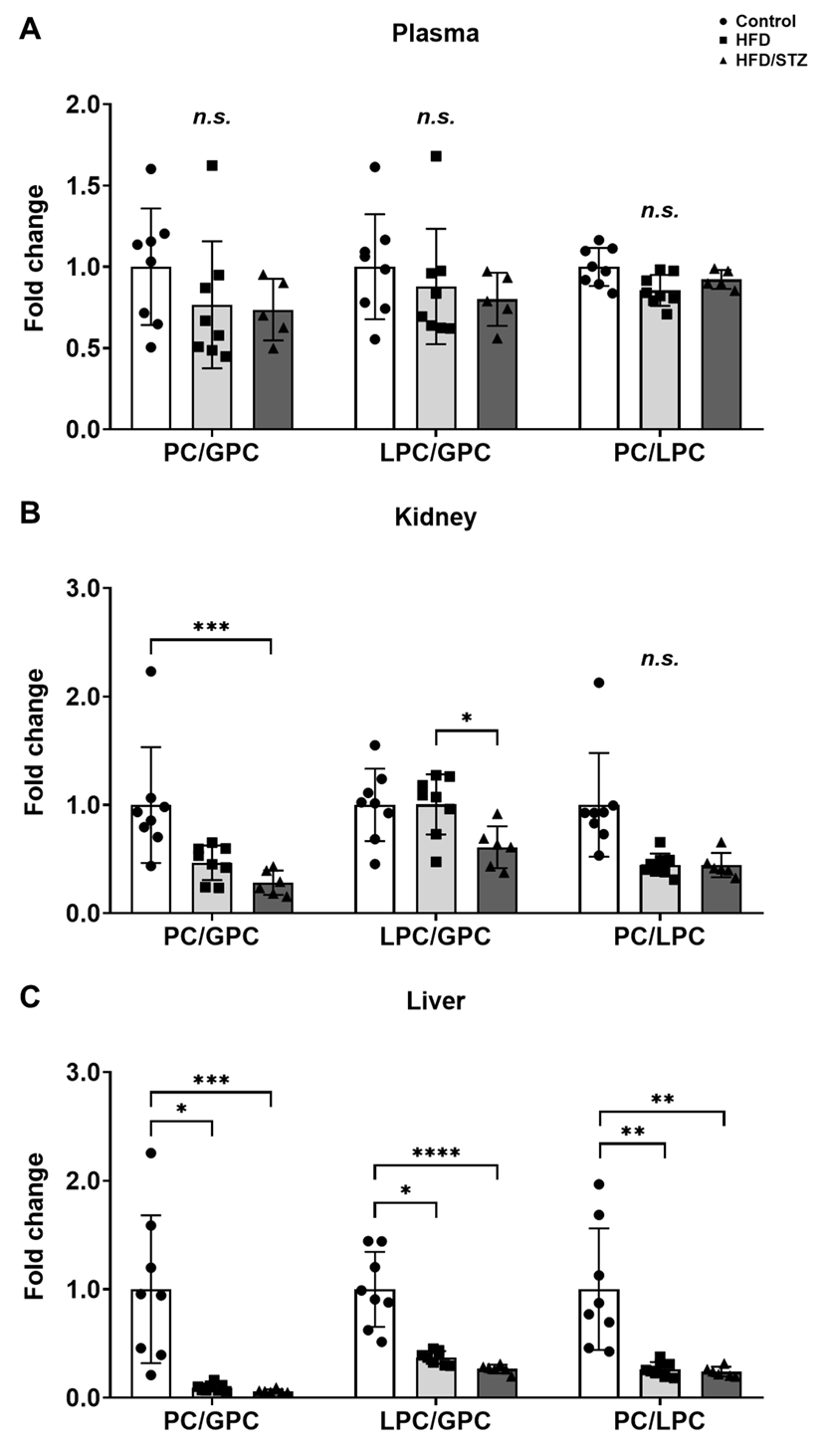


**Supplementary Figure 4**. Alterations in the ratio of PC metabolites in the plasma (A), kidney (B), and liver (C) samples (mean ± standard deviation). **p* < 0.05, ***p* < 0.01, ****p* < 0.001, *n.s*., not significant. GPC: glycerophosphorylcholine; HFD: high-fat diet; LPC: lysophosphatidylcholine; PC: phosphatidylcholine; STZ: streptozotocin.
